# Nanostructured Lipid Carrier-Based Codelivery of Raloxifene and Naringin: Formulation, Optimization, In Vitro, Ex Vivo, In Vivo Assessment, and Acute Toxicity Studies

**DOI:** 10.3390/pharmaceutics14091771

**Published:** 2022-08-25

**Authors:** Abdulsalam Alhalmi, Saima Amin, Zafar Khan, Sarwar Beg, Omkulthom Al kamaly, Asmaa Saleh, Kanchan Kohli

**Affiliations:** 1 Department of Pharmaceutics, School of Pharmaceutical Education and Research, Jamia Hamdard, New Delhi 110062, India; 2 School of Pharmacy & Biomedical Sciences, University of Central Lancashire, Flyde Road, Preston PR1 2HE, UK; 3 Department of Pharmaceutical Sciences, College of Pharmacy, Princess Nourah bint Abdulrahman University, P.O. Box 84428, Riyadh 11671, Saudi Arabia; 4 Lloyd Institute of Management and Technology (Pharm.), Plot No 11, Knowledge Park-II, Greater Noida 201308, India

**Keywords:** raloxifene, naringin, acute toxicity study, combination, nanostructured lipid carriers, central composite design, breast cancer

## Abstract

This work aimed to develop dual drug-loaded nanostructured lipid carriers of raloxifene and naringin (RLX/NRG NLCs) for breast cancer. RLX/NRG NLCs were prepared using Compritol 888 ATO and oleic acid using a hot homogenization–sonication method and optimized using central composite design (CCD). The optimized RLX/NRG NLCs were characterized and evaluated using multiple technological means. The optimized RLX/NRG NLCs exhibited a particle size of 137.12 nm, polydispersity index (PDI) of 0.266, zeta potential (ZP) of 25.9 mV, and entrapment efficiency (EE) of 91.05% (raloxifene) and 85.07% (naringin), respectively. In vitro release (81 ± 2.2% from RLX/NRG NLCs and 31 ± 1.9% from the RLX/NRG suspension for RLX and 93 ± 1.5% from RLX/NRG NLCs and 38 ± 2.01% from the RLX/NRG suspension for NRG within 24 h). Concurrently, an ex vivo permeation study exhibited nearly 2.3 and 2.1-fold improvement in the permeability profiles of RLX and NRG from RLX/NRG NLCs vis-à-vis the RLX/NRG suspension. The depth of permeation was proved with CLSM images which revealed significant permeation of the drug from the RLX/NRG NLCs formulation, 3.5-fold across the intestine, as compared with the RLX/NRG suspension. An in vitro DPPH antioxidant study displayed a better antioxidant potential of RLX/NRG in comparison to RLX and NRG alone due to the synergistic antioxidant effect of RLX and NRG. An acute toxicity study in Wistar rats showed the safety profile of the prepared nanoformulations and their excipients. Our findings shed new light on how poorly soluble and poorly permeable medicines can be codelivered using NLCs in an oral nanoformulation to improve their medicinal performance.

## 1. Introduction

**Figure 1:**
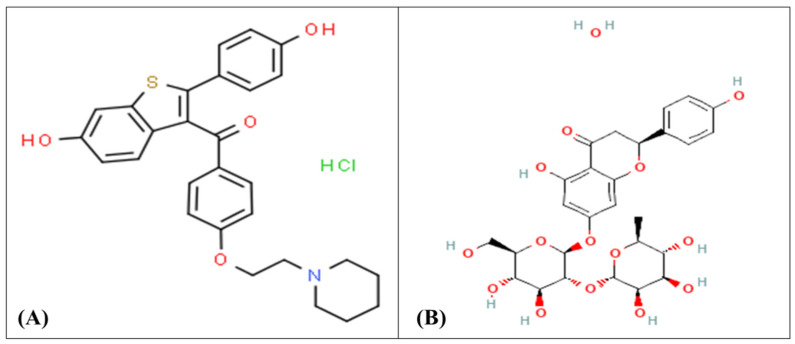
The chemical structure of (**A**) raloxifene and (**B**) naringin.

Breast cancer has long been established as one of the deadliest diseases and represents a major threat to women’s life expectancy worldwide [1]. Estrogen receptor-positive (ER+) breast cancer is the most common type of progressive breast cancer in women, and its progression is facilitated by the estrogen hormone which regulates the levels of cyclin D1, Bcl-2, Myc, and VEGF, all of which are required for cell cycle, cell survival, and angiogenesis stimulation [2,3]. Recently, various strategies for treating breast cancer have emerged, including antiestrogenic therapy with estrogen receptor modulators such as raloxifene hydrochloride (RLX) (Figure 1A), which is specifically used for (ER+) breast cancer. Since 1997, RLX has been used to treat postmenopausal osteoporosis [4]. It later received FDA approval recommendation to lower the risk of invasive breast cancer in postmenopausal women with osteoporosis and as a breast cancer chemopreventive therapy [5]. Mechanistically, RLX acts by binding to estrogen receptors, causing a conformational change in the receptors and, as a result, a change in the expression of estrogen-dependent genes [6].

Naringin (NRG) (Figure 1B) is a potent natural antioxidant flavonoid that has shown a broad important in vitro anticancer activity against various cancers of the stomach, pancreas, intestines, liver, cervix and leukemia through regulation of multiple inhibition pathways [7,8]. In an in vitro cell line study of MCF-7 breast cancer, it exhibited inhibitory effects on the phosphorylation of extracellular signal-controlled protein kinase 1 and 2 (ERK1/2) and protein kinase B (AKT) [9,10]. NRG treatment of the MCF-7 cell line boosted caspase-3 and -8 activation and promoted apoptosis via upregulation of proapoptotic genes and downregulation of antiapoptotic genes [11]. In addition, NRG and its active metabolite naringenin have been identified as xenoestrogens; they have a structure that mimics the natural steroid hormone estrogen which may enable them to bind with ERs as agonists or antagonists [12,13,14]. Thus, it can prevent estrogen-dependent breast cancers through its antiestrogenic activities [15]. NRG therapy also inhibited the growth of breast tumors and reduced tumor weight in rats induced with 7,12-dimethylbenz[a]anthracene (DMBA) [16].

To date, various studies have proven that combination therapy is emerging as an important approach to boost the therapeutic impact and minimize the adverse effects of chemotherapeutics. Recently, most researchers focus on the combination of phytochemicals with chemotherapeutic drugs to synergize the therapeutic effects, enhance the pharmacokinetic profiles, overcome MDR, and sensitize cancer cells to chemotherapy agents [17,18]. A study by Jabri et al. discovered that coencapsulating chemotherapeutic agent paclitaxel with NRG in mixed polymeric micelles increased their internalization and in vitro cytotoxicity against MCF-7 breast cancer cells [19]. Furthermore, NRG acts as a chemosensitizer, enhancing the lethal impact of paclitaxel in prostate cancer cells synergistically [20].

Nevertheless, a combination of RLX and NRG has never been administered before. Despite the possible benefits of RLX and NRG in coadministration, some problems may arise. Firstly, RLX and NRG are both extremely hydrophobic drugs that are difficult to deliver in sufficient quantities via the oral route [21,22]. Secondly, both RLX and NRG have a strong first pass effect in the liver through glucuronidation, which means that they have low bioavailability of 2% and 9%, respectively [23,24]. Moreover, NRG has low solubility in water (MW, 598.5 g/mol; log P, −0.44; TPSA, 226 Å²), which is slightly higher than that of RLX (MW, 510 g/mol; log P, 5.69; TPSA, 98.2 Å²). Both RLX and NRG belong to the class II category in the BCS classification [25,26].

Therefore, novel nanocarriers are needed to overcome such problems to improve the bioavailability and the capacity to load the two drugs in a sufficient amount. In this context, several nanocarriers have been designed for combination delivery and suggested as a novel and promising strategy in cancer treatment [27,28]. Recently, nanostructured lipid carriers (NLCs), which are lipid-based nanoparticles that include solid and liquid lipids, have been explored as a potential carrier vehicle to solve the shortcomings of previous nanoformulation platforms, such as solid lipid nanoparticles, nanoemulsions, and liposomes, in terms of improved shelf life, high bioavailability, sustained drug release, and large-scale production [29]. Moreover, to overcome the disadvantages of SLNs such as expulsion of the drug during storage and poor drug-loading capacity, NLCs were introduced as a more advanced carrier system [30]. The inclusion of liquid lipids with solid lipids in NLCs (not included in SLNs) results in imperfections in the lipid matrix. These imperfections in the lipid matrix prevent the leakage of the drug during long-term storage and result in a higher drug payload, which is the main reason behind the selection of NLCs over SLNs [31]. Importantly, solid and liquid lipids of NLCs are more likely to accommodate drugs than solid lipids or liquid lipids alone [31]. Drug bioavailability could be enhanced by lipid-based delivery, which enhances transport through the intestinal epithelium and protects the drugs from the extreme environment of the GI tract [32]. In addition, NLCs allow drugs to be targeted via the lymphatic system, resulting in several benefits such as protection from hepatic first pass metabolism, reduced hepatotoxicity, and enhanced drug bioavailability [33,34].

In this work, we aimed at developing stable NLCs to coload RLX and NRG simultaneously using a homogenization–ultrasonication method. To our knowledge, no prior study has detailed the usage of coloaded nanocarriers in this manner. Herein, RLX/NRG NLCs were successfully fabricated and optimized through the use of central composite design (CCD) with systemic characterizations for particle size, zeta potential, entrapment efficiency (EE%), SEM, TEM, XRD, FTIR, in vitro drug release, in vitro antioxidant, and stability studies. Moreover, ex vivo permeation and acute toxicity studies were performed in healthy rats. The combination of anticancer drugs such as RLX with naturally bioactive molecules such as NRG would be a unique way to improve the therapeutic efficacy of breast cancer management and treatment.

## 2. Materials and Methods

### 2.1. Materials

Raloxifene hydrochloride (RLX) was provided by Aarti Drugs Ltd. (New Delhi, India) while naringin hydrate (NRG) and KBr were obtained from Tokyo Chemical Industry Co., Ltd. (Tokyo, Japan). Labrasol^®^, Labrafac^®^, Precirol ATO5^®^, Transutol^®^, Labrafil^®^, Caproyl 90^®^, Capmul MCM^®^, Gelucire 50/13^®^, Gelucire 48/16^®^, and Compritol 888 ATO^®^ were generously provided by Gattefosse (Saint-Priest, France). Soyabean oil, peanut oil, sesame oil, corn oil, glyceryl monostearate, and sunflower oil were purchased from Loba Chemie (Chennai, India). Methanol, acetonitrile, ethyl oleate, PEG 400, Tween 80^®^, D-mannitol, methylene chloride, and hydrochloric acid were received from Central Drug House Pvt. Ltd. (Chennai, India). Almond oil, olive oil, castor oil, and 2,2-diphenyl-1-picrylhydrazyl (DPPH) were obtained from SRL Pvt. Ltd. (Chennai, India). Oleic acid, rhodamine B, and stearic acid were received from Sigma-Aldrich (New Delhi, India). The other materials utilized in this work were of standard analytical quality and used as received from their commercial source.

### 2.2. Methods

#### 2.2.1. Excipients Screening

An essential preformulation step in the fabrication of successful NLCs is selection of appropriate excipients [35]. We consider the solubility of RLX and NRG in different liquid oils while choosing a liquid lipid for the RLX/NRG NLCs formulation. In this context, an extra quantity of RLX and NRG was added to 2 mL of a chosen oil (viz., oleic acid, sesame oil, ethyl oleate, sunflower oil, rose oil, Capryol 90, corn oil, olive oil, almond oil, castor oil, Labrafac, Capmul MCM) in small glass vials and then mixed briefly on a vortex mixer (Sphinix Pvt. Ltd., Mumbai, India), followed by 72 h of stirring at 25 °C in a mechanical shaker (Grower Enterprises, Chennai, India). Following that, the tested samples were spun at 12,000 rpm for half an hour using a high-speed centrifuge (Remi Pvt. Ltd., Mumbai, India) before being analyzed. Thereafter, 200 µL from the supernatant layer were collected and diluted in 5 mL methanol and estimated for RLX and NRG quantity using a spectrophotometric apparatus (1700-UV, Shimadzu Corporation, Kyoto, Japan) at 289 nm and 284 nm, respectively. Vierordt’s method, or simultaneous equation method, was developed and validated for the simultaneous estimation of RLX and NRG. The linearity of the calibration plots was confirmed by the high value of the correlation coefficients (R^2^ = 0.9994 for RLX and 0.995 for NRG). The limit of detection and the limit of quantification were theoretically calculated and found to be 0.57 µg/mL and 0.613 µg/mL and 4.25 µg/mL and 4.55 µg/mL for RLX and NRG, respectively.

Likewise, RLX and NRG solubility tests were conducted in various solid lipids (Compritol ATO 888, GMS, stearic acid, Gelucire 50/13, Gelucire 48/16, and Precirol ATO 5) to find the best solid lipid suitable to load the maximum quantity of RLX and NRG in the NLC formulation. Briefly, we took a known quantity of both drugs (5 mg) and placed it separately in glass vials, to which 50 mg of each solid lipid were added individually. The blender with the lipid and RLX or NRG was heated in a reciprocally agitated water bath to a temperature that was 10 °C above the melting temperature of the solid lipid. Following that, 50 mg of the solid lipid at a time were added until 5 mg of the drugs were entirely dissolved and the molten lipid formed a transparent dispersion [36]. Through the use of various solid-to-liquid lipid ratios, we were able to determine if the optimized binary lipid combination was physically compatible. The binary mixtures were melted with mixing for 1 h and then cooled down to room temperature before being visually examined in front of a white sheet to observe the physical integrity of the lipid mixture. The binary mixture of the selected lipids that did not disclose any phase separation was selected for the fabrication of NLCs for loading RLX and NRG. The surfactants were chosen based on their ability to emulsify the optimal binary mixture of solid lipids and liquid lipids. Surfactants (Tween 60, Tween 80, Transcutol, Labrasol, and Span 20) were used for the screening. A weighted quantity of 300 mg of the optimized binary lipid mixture in a test tube was dispersed by addition of 20 mL of methylene chloride and stirring at room temperature. Thereafter, we added 10 mL of an aqueous surfactant solution (5% w/v) to the dispersion with shaking. Following that, the solution was heated to 50 °C for 20 min or till all the methylene chloride was evaporated. Thereafter, 2 mL of this solution was blended with 10 mL of double-distilled water, and the % transmittance (% T) at 517 nm was estimated utilizing a UV spectrophotometer [37]. Furthermore, it was essential to consider the solubility profiles of the drugs in the surfactants and the HLB values of the surfactants while choosing surfactants.

#### 2.2.2. Experimentation Design

In this research, the design and optimization of RLX/NRG NLCs were carried out using central composite design (CCD) (Design Expert^®^ software, version 13, Stat-Ease, Minneapolis, MN, USA). The interest response can be investigated using CCD evaluation that measures how much influence it has on process variables. As an outcome, the number of research experiments that are necessary to identify a mathematical pattern in the experimental design is greatly decreased, allowing for evaluating the variable elements and their optimal level required for a specific response [38]. Here, several preliminary tests in the process formulation were conducted to identify the processing factors that displayed a statistically significant influence on the outcomes. The amount of lipids (solid lipids and liquid lipids) (mg) (A), the amount of surfactants (mg) (B), and sonication time (min) (C) were the process variables (independent variables) that were determined from the preliminary experiments, while the RLX and NRG quantities and ratios of solid lipids to liquid lipids, as well as the ratio of Tween 80 to Labrasol were all deemed fixed values. In this study, as shown in Table 1, three-level and three-factor CCD was used for screening and optimization of the process variables for preparation of RLX/NRG NLCs and to test the effects of variation of process variables on the dependent variables (responses), which include the particle size (Y1), PDI (Y2), entrapment efficiency of RLX (EE%) (Y3), and entrapment efficiency of NRG (EE%) (Y4). Twenty different designs of the runs were performed to choose the appropriate model and limit the experimental error. Using a correlation and optimization technique, three-dimensional (3D) surface response plots were evaluated for statistical correlation and optimization of various factors and their interaction with relation to the responses obtained. Moreover, variance analysis (ANOVA) was used in this study to evaluate the statistical relevance of each model coefficient.

#### 2.2.3. Formulation of RLX/NRG NLCs

NLCs loaded with both RLX and NRG (RLX/NRG NLCs) and blank NLCs (without RLX and NRG) were made by hot homogenization with probe sonication as reported previously [39] with some alterations. Briefly, solid lipid Compritol 888 ATO and liquid lipid oleic acid (total of 200–400 mg) at a 3:1 ratio were mixed and heated with stirring till melting at 75–80 °C, and NRG and/or RLX (20 mg and/or 10 mg, respectively) were incorporated into the lipid phase. To create the aqueous phase, Tween 80 and Labrasol (100–200 mg at a fixed weight ratio of 1:1) were mixed in double-distilled water (20 mL), which was preheated to 75–80 °C. Subsequently, the surfactant aqueous phase was gently added dropwise to the lipid phase and homogenized at 1200 rpm with continuous heating for 20 min. Using a probe sonicator, the pre-emulsion was further sonicated (work time, 20 s; rest time, 5 s) for 1–3 min, allowing it to settle down to room temperature while being gently agitated. A similar procedure was used to produce blank NLCs, RLX NLCs, and NRG NLCs.

#### 2.2.4. Lyophilization of RLX/NRG NLCs Formulations

To lyophilize the RLX/NRG NLCs, a cryoprotectant comprising 2% (w/v) pure D-mannitol was incorporated. Briefly, RLX/NRG NLCs were frozen in a Petri dish for 24 h at −20 °C and the frozen samples were dried under vacuum in a freeze-dryer (Lyodel, Delvac Pumps Pvt. Ltd., Mumbai, India) for dryness up to 30 h.

#### 2.2.5. In Vitro Characterization of RLX/NRG NLCs

##### X-ray Diffraction (XRD)

The X-ray diffractometer (6000-XRD, Shimadzu, Tokyo, Japan) was used to characterize the crystalline structure of RLX, NRG, Compritol 888 ATO, the physical mixture, lyophilized blank NLCs, and lyophilized RLX/NRG NLCs. Cu Kα radiation (40 kV; 40 mA) was applied to the powder samples at a scan rate of 0.02°/s over a 2 min range of 10–80° [40].

##### Fourier-Transform Infrared Spectroscopy (FTIR)

FTIR spectrum of pure (RLX, NRG, Compritol 888 ATO, and D-mannitol) and lyophilized materials (blank NLCs and RLX/NRG NLCs) was generated using the KBr press method and conducted on an IR spectrophotometer (Shimadzu Corp, Kyoto, Japan). The tested powder samples were triturated with 50 times their weight in potassium bromide and pressed into small pellets using a mini-press under extremely high pressure (3000 psi). The spectra were obtained in the 400–4000 cm^−1^ wave range [41].

##### Microscopic Evaluation

RLX/NRG NLCs’ morphological surface of the optimized formulations evaluated by transmission electron microscopy (TEM) (Tecnai G20 HR-TEM, Thermo Scientific, Waltham, MA, USA) and scanning electron microscopy (SEM) (Zeiss EVO 18, Oberkochen, Germany, Gemini 5, and Germany). The RLX/NRG NLCs were previously diluted at 1:100 with distilled water before use. For TEM analysis, three drops of the diluted sample were dispersed on the surface of carbon-coated copper grids (300 mesh); the sample was stained with a few drops of 2% (w/v) phosphotungstic acid and then kept to dry overnight at room temperature. The dried residue of the sample was displayed under TEM. For SEM analysis, three drops of RLX/NRG NLCs were placed on a double-sided carbon tape mounted on an aluminum stud, then vacuum-coated with gold for 5 min. The dried sample of the optimized RLX/NRG NLCs was displayed under SEM [42].

##### Particle Size, Polydispersity Index, and Zeta Potential Measurement

The intensity average of particle size, PDI, and zeta potential of the optimized RLX/NRG NLCs were analyzed using the dynamic light scattering principle with a zeta-sizer device (Malvern Zeta-Sizer, Worcestershire, UK) [43]. Before the measurements, all of the tested samples had been dispersed with 50 folds of Milli-Q water to create a 2% uniform dispersion. All the obtained values are the means of three measurements.

##### Entrapment Efficiency (EE)

The EE of RLX/NRG NLCs was estimated by separation of un-entrapped RLX and NRG from RLX/NRG NLCs using the centrifugation process as described in [44]: 2 mL of the NLCs loaded with drugs were centrifuged for 40 min at 15,000 rpm using a high-speed centrifuge (Beckman Coulter India Pvt. Ltd., Mumbai, India). Subsequently, separation of the supernatant layer was carefully followed by proper methanol dilution and filtration through a syringe membrane filter (0.45 μm). Then, the filtrated solution was subjected to drug estimation for free RLX and NRG spectrophotometrically (Shimadzu, Japan). The EE was computed using the following equation:

(1) $$\mathrm{Entrapment}\;\mathrm{efficiency}\;\left(\mathrm{EE}\%\right)=\frac{\;{\left[D\right]}_{\;\left[\mathrm{Total}\right]}-{\left[D\right]}_{\left[\mathrm{Free}\right]}\;}{{\left[D\right]}_{\left[\mathrm{Total}\right]}}\times 100$$

where [D]_Total_ is the initial weight of the RLX and NRG added to the formulation and [D]_Free_ is the weight of free unloaded RLX and NRG detected in the supernatant layer. Therefore, we consider that all the RLX and NRG not present in the supernatant layer were successfully loaded in the lipid matrix of the NLCs [45].

##### In Vitro Drug Release and Kinetics Modeling

To conduct in vitro drug release studies, the activated dialysis tube membrane system (12 KDa, Himedia Ltd., New Delhi, India) was employed [46]. In short, a weighted quantity of drug-containing RLX/NRG NLCs and the RLX/NRG suspension was inserted in the dialysis tube and dipped into 80 mL dissolution media (0.1 N HCl, pH 1.2, for 2 h) and phosphate buffer (6.8 g of KH_2_PO_4_ and 0.94 g of NaOH dissolved in 1 L distilled water to give pH 6.8, for the remaining 22 h). An amount of 0.2% v/v Tween 80 was also added to the dissolution medium to ensure the sink condition. After a specified period, the samples (5 mL) were extracted and immediately replaced with an equal amount of fresh dissolution medium to maintain the sink condition. The samples filtered were later tested using a validated UV spectrophotometric technique (Vierordt’s method) (UV-1700, Shimadzu, Tokyo, Japan). The data were shown as a cumulative percentage of RLX and NRG release with respect to time.

Released (D) (%) = FR/IDC × 100(2)

where FR = fraction release of RLX or NRG to the external medium and IDC = initial drug concentration of RLX or NRG inside the dialysis tube. Using the cumulative release data, release kinetics modeling was carried out, and the kinetic model with a higher value of the correlation coefficient (R^2^) was selected as the best-fitting model [47].

##### In Vitro Antioxidant Activity

The radical scavenging potential of the ethanolic solution of RLX, NRG, RLX/NRG, and RLX/NRG NLCs were evaluated by radical DPPH and compared to that of a standard ascorbic acid solution. The evaluation was based on the DPPH’s potential activity in scavenging free radicals. Briefly, the activity of DPPH as a radical scavenger was determined using a modified technique developed by Kumar et al. [48]. A 2 mL solution of 0.04% w/v solution of DPPH in ethanol was added to 2 mL of various concentrations of RLX, NRG, RLX/NRG, and RLX/NRG NLCs, and the reaction mixture was kept for 45 min in a dark area at 25 °C. UV spectrophotometric measurements were taken at 517 nm to determine the absorbance of the tested samples and the DPPH solution. The DPPH scavenging capacity percentage was determined using Equation (3) and taking absorbance of DPPH as a control.

(3) $$\mathrm{Inhibition}\;\mathrm{of}\;\mathrm{DPPH}\;\mathrm{radical}\;\%=\frac{\mathrm{Absorbance}\;\left(A\right)-\mathrm{Absorbance}\;\left(B\right)}{\mathrm{Absorbance}\;\left(A\right)}$$

where (A) refers to the blank absorbance (control) and (B) refers to the sample absorbance. The graph of the inhibition of DPPH percentage versus sample concentration was plotted, followed by the determination of the inhibitory concentration percentage (IC50) by interpolation of data and then comparison with the IC50 of the standard ascorbic acid [49].

### 2.3. Animal Studies

Albino female Wistar rats (200–250 g) used in this project were preapproved by the Institutional Animal Ethics Committee, Jamia Hamdard, India (approval No. IAEC-70-JH-1784/CPCSEA, dated 8 April 2021). All the animals were housed in the animal house facility of Jamia Hamdard in polypropylene cages under recommended laboratory conditions with proper and timely cleaning and with a standard diet.

#### 2.3.1. Ex Vivo Permeability Study

The intestinal non-everted rat sac model was utilized for the ex vivo intestinal uptake analysis [50]. Shortly, female Wistar rats (200–250 g) fasted overnight got anesthetized with a combination of ketamine and xylazine, were sacrificed, and thereafter, the intestine was excised and the ileum segment was removed and thoroughly rinsed with Ringer’s solution to eliminate all waste elements. Subsequently, weighted amounts of the tested samples containing RLX/NRG NLCs and the RLX/NRG suspension were inserted separately into the intestinal sac. The sac was then tightened from both ends and dipped into a jacket glass containing dissolution media (Tyrode’s buffer (40 mL)), pH = 6.8, prewarmed to 37 ± 0.5 °C and supplied by an aerator with 95% oxygen for 2 h. At specified time intervals, an aliquot (2 mL) was taken, and the sink condition was maintained. The sample was diluted and filtered and then analyzed using a validated UV spectrophotometry method [51]. The quantity of the drugs that permeated out of the sac within 120 min was estimated. The apparent permeability (P_app_) was estimated as a quantitative indication of the rate at which the drugs were transported across the intestinal membrane per unit of time (cm/min) and calculated using the equation below:

(4) $$P_{\mathrm{app}}=(\mathrm{dQ}⁄\mathrm{dt})/A\;\times \;\mathrm{Ci}$$

where dQ/dt is the rate of drug penetration or permeation flux (F) (µg/min) determined from the slope of the linear curve by plotting the cumulative quantity of the drugs internalized in the intestinal sac over time, Ci is the starting amount of a drug within the mucosal sac (µg/mL), and A is the total sac’s surface area (7.46 cm^2^) [52].

#### 2.3.2. Confocal Laser Scanning Microscopy (CLSM)

Rhodamine B-labeled RLX/NRG NLCs and RLX/NRG suspension were prepared by incorporating rhodamine B (0.02% w/v) during the formulation process and used for intestinal uptake assessment. Female Wistar rats fasted for 24 h and were sacrificed to obtain a 2.36-inch-long part of the ileum, which was cleaned with Tyrode’s buffer to remove fecal material. The samples were inserted into a separate sac and tightly closed from both ends and mounted in Tyrode’s buffer for 3 h (at 37 ± 2 °C, 45 rpm, and 95% O_2_ supply). The sacs were then washed with Tyrode’s buffer to remove excess free rhodamine B, cut into small species longitudinally, and placed into slits [53]. The depth penetration of the rhodamine B-labeled drug from the formulation and the suspension across the intestinal wall was detected and analyzed for the z-axis by CLSM (Leica Microsystems SP8, Mumbai, India).

#### 2.3.3. In Vivo Acute Oral Toxicity

To evaluate the pharmacological safety of the prepared NLC nanoformulations, acute repeated toxicity studies were carried out by administering the tested drug-loaded nanoformulations at their therapeutic dose [54]. This study was carried out on female Wistar rats following the guidelines of the Organization for Economic Co-operation and Development (OECD). The rats were randomly selected and assigned to the following five test groups (six animals per group) and treated with normal saline, blank NLCs (2 mL), RLX NLCs, NRG NLCs, and RLX/NRG NLCs at their therapeutic doses of RLX (30 mg/kg) and NRG (40 mg/kg) via the oral gavage route daily for 14 days. Throughout the study, all the animals were monitored daily for clinical indicators of toxicity, mortality, and behavioral abnormalities, as well as changes in physical appearance, injury, pain, and signs of sickness. Bodyweight was recorded during pretreatment and during the post-treatment period. On the 15th day, the rats were anesthetized with ketamine–xylazine (75–10 mg/kg body weight i.p.), and blood samples were collected via a heart puncture into EDTA tubes, as previously described [55]. The collected blood was used for hematological analysis. Then, the rats were euthanized and the vital organs (liver, heart, and kidneys) were collected, cleaned in normal saline, and placed in 10% formalin; then, the samples were dehydrated with ethanol and embedded in paraffin blocks; thereafter, the tissues were mounted on glass slides and cut into thin sections of around 6 µm using a rotary microtome (Leica Multicut 2045, Reichert-Jung Products, Wetzlar, Germany). After histological staining with hematoxylin and eosin (HE), the slides were studied using a light microscope (ZEISS Primostar, Oberkochen, Germany) to monitor for any significant morphological changes. Photomicrographs of tissue sections from the test and control groups were recorded by a consultant histopathologist who documented the pathological differences in histological sections.

### 2.4. Storage Stability

To determine the storage stability of the final RLX/NRG NLCs, the optimized lyophilized RLX/NRG NLCs were subjected to accelerated storage conditions for three months. The lyophilized samples were stored at 4 °C, 25 °C, 2 °C/60% RH, and 40 °C, 2 °C/75% RH in amber-colored containers in a stability chamber (Macro Scientific, Mumbai, India). Samples were obtained and analyzed for particle size, EE%, and redispersibility at predefined intervals (0, 1, 3, 6, 9, and 12 weeks) [56].

### 2.5. Statistical Analysis

All the data are presented as the means ± standard deviation (SD) of three replicates. Graph Pad Prism Software was utilized for the statistical analysis (Instat 3.06, San Diego, CA, USA). For the statistical analysis of various parameters in the optimization part, Design Expert^®^ (Version 13, State-Ease Inc., Minneapolis, MN, USA) was employed. *p*-values < 0.05 were regarded to be significant.

## 3. Results and Discussion

### 3.1. Excipients Selection

**Figure 2:**
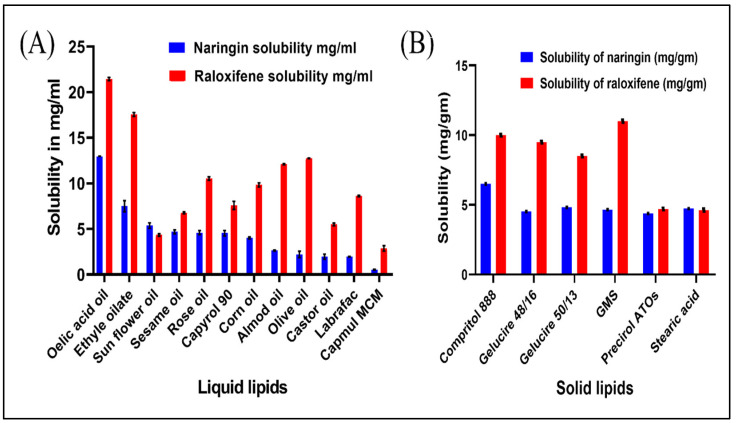
Solubility profiles of RLX and NRG in (**A**) liquid lipids (at 25 °C) and (**B**) solid lipids.

Encapsulation efficiency (EE) of RLX/NRG NLCs is highly dependent on the RLX and NRG solubility profiles in a lipid matrix. It was revealed that RLX and NRG have different solubility profiles depending on whether they are dissolved in liquid or solid lipids. The decreasing order of solubility of RLX and NRG in various tested liquid lipids and solid lipids is displayed in Figure 2A,B. Oleic acid was chosen as a liquid lipid because of the high solubility of both RLX and NRG in it, which was found to be 21.43 ± 1.58 mg/mL for RLX and 12.597 ± 0.95 mg/mL for NRG. The high solubility of RLX and NRG in oleic acid might be due to its natural self-emulsifying property; thus, oleic acid was selected as the liquid lipid. The solubility of RLX was as follows: GMS > Compritol 888 ATO > Gelucire 48/16 > Gelucire 50/13 > Precirol > stearic acid (11, 10, 9.5, 8.5, 4.69, and 4.39 mg/gm, respectively), while the solubility of NRG was as follows: Compritol 888 ATO > Gelucire 50/13 > stearic acid > GMS > Gelucire 48/16 > and Precirol (6.5, 4.8075, 4.725, 4.643, 4.52, and 4.37 mg/gm, respectively). Primarily, solid lipid GMS was selected as the maximum solubility of RLX in it was found to be 11 ± 0.35 w/w, while in the case of NRG, the maximum solubility was in GMS (4.643 ± 0.51 w/w), but it was not selected as we took into consideration NRG solubility was not maximum. Compritol 888 ATO exhibited a higher solubilizing capability for RLX and NRG, which can be associated with the existence of long-chain fatty acids, and was thus chosen as the solid lipid for the formulation of RLX/NRG NLCs.

In accordance with the findings of the miscibility test performed on the binary combination, it was revealed that the mixture of Compritol 888 ATO and oleic acid demonstrated acceptable miscibility without phase separation at various ratios (Supplementary Table S1). The binary blend of Compritol 888 ATO and oleic acid in a 3:1 ratio was adopted as a midpoint for the formulation of RLX/NRG NLCs to realize the largest anticipated solubility of RLX and NRG. Moreover, it was critical to examine the capacity of surfactants to emulsify the dispersion of binary lipids while choosing them. The capacity of surfactants to emulsify was determined using transmittance percentage: Labrasol (95.1 ± 0.73), Tween 80 (79.43 ± 0.6), Transcutol (64.40 ± 0.40), Tween 60 (49.3 ± 0.50), and Span 20 (32.20 ± 0.57). As a result, the Labrasol and Tween 80 solutions showed the greatest transmittance % and emulsification capacity and were chosen as the optimum combination in a one-to-one ratio for enhancing emulsification outcomes. Furthermore, previous studies showed that Labrasol and Tween 80 block the function of P-gp, causing intestinal absorption of P-gp substrates to increase [57,58].

### 3.2. Experimentation Design

**Figure 3:**
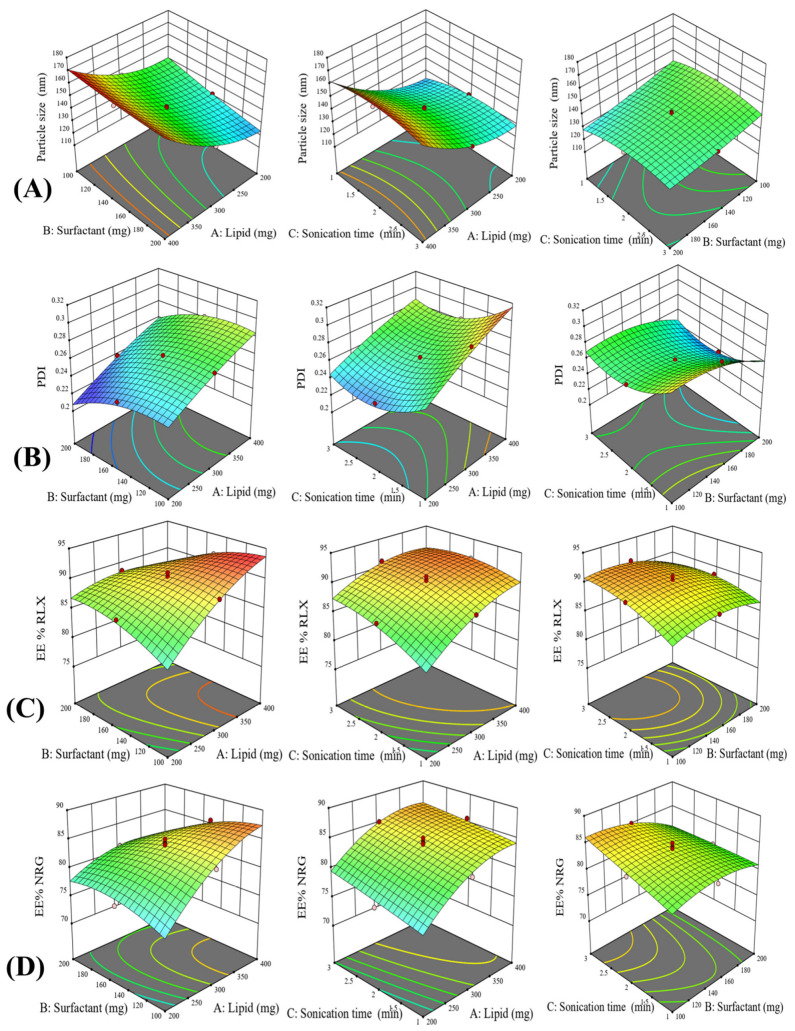
Three-dimensional response surface plots demonstrating the effect of variation in process variables on (**A**) particle size, (**B**) PDI, and (**C**,**D**) entrapment efficiency of RLX/NRG NLCs.

The formulation of nanostructured lipid carriers for codelivery of drugs is a complicated procedure that involves many processing factors. These factors exhibit a significant impact on their individual form or in their interaction among themselves which is finally reflected in the quality of the final formulations. The process parameters and their level used in the experiments are mentioned in Table 2. An experimental design of 20 runs containing five centerpoints was made according to the CCD mathematical design for these chosen parameters to define the optimal amounts of the process parameters affecting different responses. Through performing the procedure at varying levels of all variables, the individual and interactive effects of process variables were observed. All the responses found in all the runs were concurrently adapted to various models using the Design Expert 13 software. The quadratic model was shown to be the best-fitting model for determining the association between significant factors and response. Experimental data results and simulation values are listed in Table 2. Based on the coefficient of correlation (R2) values for analyzing the impacts of independent variables on responses, it was concluded that the quadratic model was the best-fitting model among the several models studied. The summary statistics for regression analysis of the responses Y1, Y2, Y3, and Y4 for data fitting into several models, including the linear, 2FI, quadratic, and cubic models, are provided in Table 3. This quadratic model resulted in several surface response plots. A few representative significant surface reaction plots of the measured model for the average particle size, PDI, and drug entrapment efficiency are shown in Figure 3. Moreover, for the particle size, PDI, and entrapment efficiency, an actual experimental values of the responses vs. the matching anticipated values plot and a perturbation plot were made using the Design Expert software to generate a scatter graph as displayed in Supplementary Figures S1 and S2.

#### 3.2.1. The Impact of Independent Variables on Particle Size

Table 2 shows the particle size distribution collected from a variety of experimental runs. It is estimated that the average particle size ranges between 115.6 and 169.02 nm. After being fitted to a range of model systems, the results revealed that the quadratic model provided the ultimate fit. The results demonstrate the model’s significance since they explain the relationship between the process variables and the obtained responses. A *p*-value of < 0.05 indicates that the model terms are significantly based on statistical evaluation of model validity (Supplementary Table S2). The statistical correlation between the input parameters and the particle size for RLX/NRG NLCs was illustrated in terms of the coded factors in the final quadratic formula and was shown to be statistically significant.

Particle size (Y1) = 140.729 + 18.039 × A + −5.528 × B + 1.388 × C + 3.325 × AB + −1.345 × AC + 1.56 × BC + 8.8745 × A^2^ + 1.0595 × B^2^ + −5.0804 × C^2^ (5)

Figure 3A depicting the 3D response surface plots for the particle size of the RLX/NRG NLCs showed that as the concentration of lipids (A) increased, the size of the RLX/NRG NLCs particles increased significantly. Likewise, the surfactant had a negative impact on particle size as stated by the coded equation, high particle size was obtained at a low level of surfactant and a low level of lipid; then, the particle size gradually decreased with an increase in the surfactant level and an increase in the lipid level to result in intermediate size at the middle point. At low lipid concentrations and intermediate levels of surfactant, smaller particle sizes were observed. One explanation for this could be the surfactant’s effect on the surface tension between the lipid phase and the aqueous phase, which leads to the production of smaller particles. Sonication time shows a positive nonsignificant impact on particle size as stated by the coded equation.

#### 3.2.2. The Impact of Independent Variables on the PDI

The PDI is responsible for the homogeneity of nanoformulations. Smaller values of the PDI were desired. According to the fit summary of the models, the quadratic model was found to be the most effective in explaining the relationship between independent variables and the PDI. As shown in Supplementary Table S3, the model’s F-value of 78.68 means that the model was statistically significant, while *p* < 0.0001 demonstrated that the model terms were statistically significant. Furthermore, the correlation coefficient (R^2^ = 0.9861) indicated a strong relationship between the process factors and the responses received. The mathematical relation between the input variables and the PDI of the RLX/NRG NLCs was demonstrated in the final quadratic formula in terms of coded factors and was proven to be statistically significant:

PDI (Y2) = 0.2619 + 0.0245 × A + −0.0142 × B + −0.011 × C + 0.001625 × AB + −0.0036 × AC + 0.0031 × BC + −0.0034 × A^2^ + −0.0109 × B^2^ + 0.0180 × C^2^ (6)

The equation expressed in terms of coded factors enables the prediction of the response for specified levels of each factor. Figure 3B displayed that the PDI of the NLCs was considerably affected by lipid content (A) with a linear increase in the PDI as the lipid level increased. Surfactant (B) and sonication time (C) had a negative impact on the PDI.

#### 3.2.3. The Impact of Independent Variables on EE% of RLX and NRG

Entrapment efficiency is an essential characteristic that plays a key part in the formulation of NLCs since the delivery of the necessary dose of the drug for therapeutic effectiveness which is integrated into NLCs is reliant on it. Several models were tested, and the quadratic model provided the best fit for the response data. The model’s F-value, lack of fit, and *p*-value all indicate that the model was statistically significant (Supplementary Tables S3 and S4).

Equations (7) and (8) represent the effect of lipid and surfactant content on the entrapment efficiency of RLX/NRG NLCs for RLX and NRG. The linear terms of lipid concentration and sonication time had a positive effect on entrapment efficiency for RLX and NRG with a coefficient value, 0.985 vs. 3.598 and 1.492 vs. 1.253, respectively, while surfactant concentration showed a negative impact on entrapment efficiency for RLX and NRG with coefficient values of −0.559 and −1.257, respectively. Interaction effects AB, AC, and BC showed a negative effect on the EE% of both drugs. As shown by the equations below, the concentration of lipids has the greatest effect on the entrapment efficiency for both drugs. As the lipid concentration increases, more drugs are entrapped, which results in a higher entrapment efficiency. In Figure 3C,D, the impact of independent factors on the entrapment efficiency of RLX and NRG in NLCs is depicted.

EE%, RLX (Y3) = 90.4334 + 2.878 × A + −0.559 × B + 1.492 × C + −2.835 × AB + −1.06 × AC + −0.785 × BC + −1.60591 × A^2^ + −1.42091 × B^2^ + −1.15591 × C^2^ (7)

EE%, NRG (Y4) = 83.913 + 3.598 × A + −1.257 × B + 1.253 × C + −2.43875 × AB + −0.35375 × AC + −1.26625 × BC + −2.285 × A^2^ + −1.53 × B^2^ + −0.12 × C^2^ (8)

The R^2^ values were 0.9886 and 0.9749 for RLX and NRG EE%, respectively, indicating a good correlation between the process variables and the responses obtained. The percentage of the entrapped drugs is generally influenced by the carrier lipid’s concentration. Compritol 888 ATO is a long-chain fatty acid (mono-, di-, and triglycerides) with an HLB value of approximately 2, which is responsible for encapsulating both lipophilic drugs, RLX and NRG. Additionally, incorporation of a liquid lipid (oleic acid) enhances drug loading by the formation of imperfect lattices in the lipid matrix [59]. On the contrary, increasing the surfactant concentration resulted in a reduction in the NLCs’ EE%. A high concentration of surfactants in the aqueous phase may cause an increase in the distribution of drugs from the lipid matrix to the aqueous medium. The entrapment efficiency values of RLX and NRG from the NLCs ranged from 77.23% to 93.32% and from 72.29% to 88.81%, respectively, and this can be explained by the higher lipophilic nature of RLX in compassion to NRG.

#### 3.2.4. Checkpoint Selection of an Optimum Formulation

**Figure 4:**
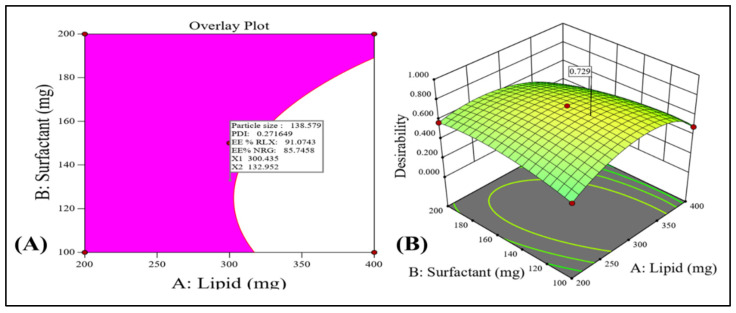
(**A**) Overlay plot generated by graphical optimization, indicating the pink area as the design space and the flagged mark as the optimized RLX/NRG NLCs. (**B**) Overall desirability value.

The best composition of the NLCs formulation is to be determined by a thorough optimization technique. According to the chosen quadratic model, the numerical and graphical optimization methodology was used with the goal of optimization of all the output parameters such as the minimum particle size for better RLX/NRG NLCs’ absorption, low values of the PDI for maintaining RLX/NRG NLCs’ homogeneity and stability, and high drug entrapment efficiency for better therapeutic activity of the RLX and NRG loaded in NLCs. Figure 4A,B shows the overlay diagram obtained throughout the graphical optimization, which displays the anticipated values of the input variables for the optimized RLX/NRG NLCs composed of a total lipid weight of 300.43 mg, a surfactant weight of 132.95 mg, and sonication time (3 min) with statistically predicted value responses, viz., particle size of 138.579 nm, PDI of 0.271 mV, RLX entrapment efficiency of 91.07%, and NRG entrapment efficiency of 85.74% with an overall desirability function value of 0.729. It was found that the prediction error was less than 5% when the anticipated and reported values were compared, proving the model’s appropriateness and accuracy.

### 3.3. Characterization of Optimized RLX/NRG NLCs

#### 3.3.1. XRD Analysis

**Figure 5:**
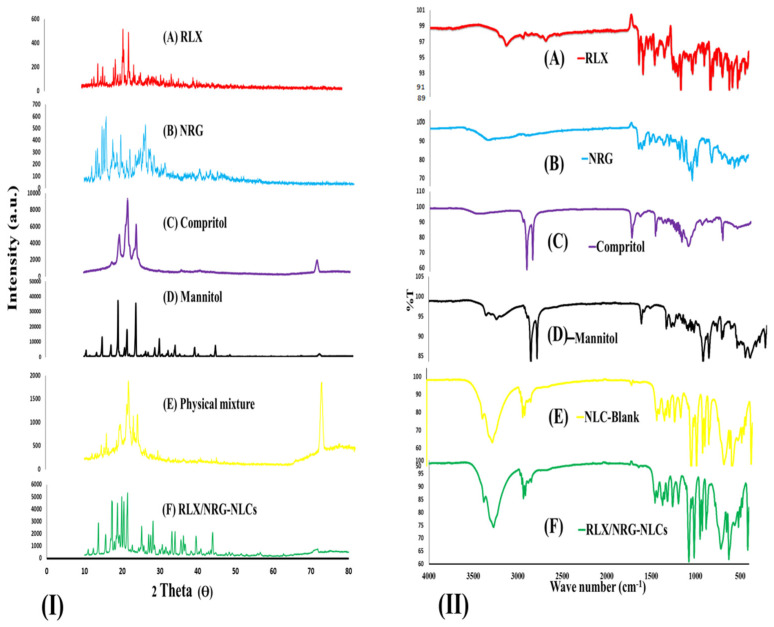
(**I**) XRD profiles of (A) raloxifene, (B) naringin, (C) Compritol 888 ATO, (D) D-mannitol, (E) physical mixture (Compritol, raloxifene, and naringin), and (F) lyophilized RLX/NRG NLCs. (**II**) FTIR spectra of (A) raloxifene, (B) naringin, (C) Compritol 888 ATO, (D) D-mannitol, (E) blank NLCs, and (F) lyophilized raloxifene/naringin nanostructured lipid carriers.

The XRD patterns of RLX, NRG, Compritol 888 ATO, D-mannitol, the physical mixture (Compritol 888 ATO, RLX, and NRG), and the lyophilized RLX/NRG NLCs are shown in Figure 5I. In Figure 5I(A,B), the diffractograms of RLX and NRG show multiple close-packed sharp peaks in the 10–30° range (2θ of 13.07, 13.49, 14.6, 15.14, 15.65, 17.38, 19.5, 21.86, 24.66, and 25.94; and 2θ of 13.07,13.49, 14.6, 15.14, 15.65, 17.38, 19.5, 21.86, 24.66, and 25.94, respectively), indicating that both drugs are highly crystalline, explaining their poor solubilities. In Figure 5I(C), Compritol’s diffractogram displays a very high and noticeable sharp peak at 2θ values 19.35, 21.51, 23.76, and 71.03, showing that lipid materials are perfectly crystalline. In Figure 5I(E), the physical mixture demonstrates all the major peaks of the drugs and the lipid in nearly the same position on the diffractogram with reduced intensities. In Figure 5I(F), the diffractogram of the lyophilized RLX/NRG NLCs is composed mostly of broad peaks, contributing to the relatively high amount of cryoprotectant mannitol and the peaks of lipid Compritol 888 ATO with a significant reduction in intensity and the absence of the numerous closely peaked sharp peaks of crystalline RLX and NRG in the diffractogram pattern of lyophilized RLX/NRG NLCs powder, which is explained by the inclusion of RLX and NRG within the lipid matrix.

#### 3.3.2. FTIR Analysis

The FTIR spectra of RLX, NRG, Compritol 888 ATO, D-mannitol, blank NLCs, and the lyophilized RLX/NRG NLCs are shown in Figure 5II. The infrared absorption spectra of pure RLX revealed characteristic absorption peaks at 3138 cm^−1^ (OH stretching), 1641 cm^−1^ (C=O stretching), 1641 cm^−1^ (C=O stretching), 1595 cm^−1^ (conjugated ketone –C–O–C stretching), 1463 cm^−1^ (S-benzothiofuran), 904 cm^−1^ (benzene ring), 806 cm^−1^ (thiophene C–H bond), 833 cm^−1^ (C–C stretching), 1122 cm^−1^ (aliphatic –C–O– stretching), and 698 cm^−1^ (–C–S– stretching), which are consistent with the previously reported values from the literature [60]. For free NRG, its characteristic peaks were shown at 3339 cm^−1^ (–OH stretching), 1642 cm^−1^ (>C=O), 1519 cm^−1^ (C=C bond of the aromatic ring), 1478 cm^−1^ (aromatic >C=C stretching), 1360 cm^−1^ (phenol C–O stretching), 1297 cm^−1^ (vibration of the −OH group), 1071 cm^−1^ (–C–O–C bonds of the ether), and 1038 cm^−1^ (cyclic –C–O stretching) as reported [61,62]. The Compritol 888 ATO spectra exhibited typical strong peaks at 2914 cm^−1^ and 2848 cm^−1^ (C–H stretching), as well as a band at 1738 cm^−1^ (C=O stretching) [63]. FTIR spectra for the RLX/NRG NLCs revealed characteristic peaks at 3274, 2937, 1458, 1431, 1371, 1316, 1261, 1193, 1067, 1018, 951, 716, and 628 cm^−1^. It can also be seen that most of the characteristic peaks of RLX and NRG disappeared when they were encapsulated into the matrix of the NLCs as revealed in Figure 5II. These observations suggest that noncovalent interactions were mainly present between the combination of the drugs and the carrier during the formation of lipid nanocarriers.

#### 3.3.3. Particle Size, PDI, Zeta Potential

**Figure 6:**
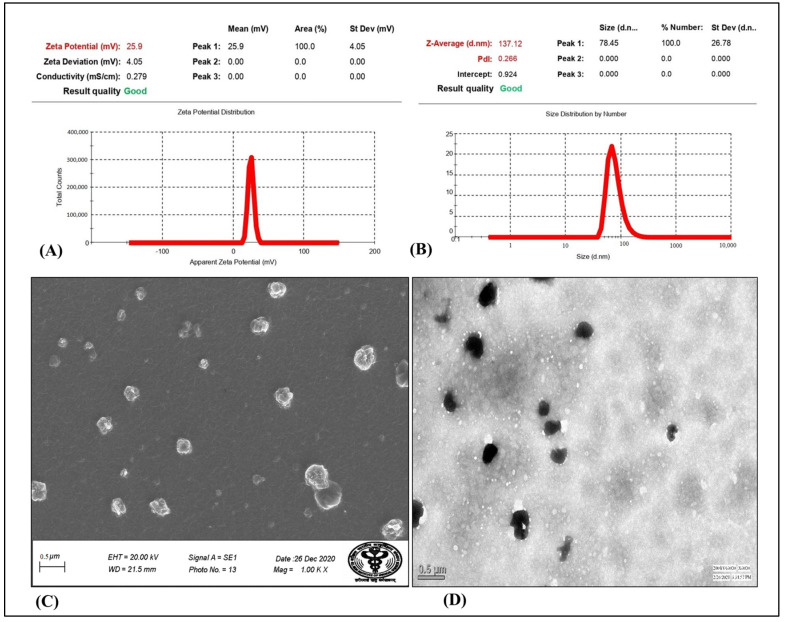
(**A**) Particle size distribution, (**B**) zeta potential, (**C**) SEM, (**D**) TEM of the optimized RLX/NRG NLCs formulation.

The assessment of particle size revealed that all of the prepared formulations had particle sizes ranging between 115.6 and 169.02 nm in size. The particle size distribution of the optimized RLX/NRG NLCs with a size of 137.12 nm and a particle size distribution index (PDI) of 0.266 are depicted in Figure 6A. The results demonstrated the monodisperse nature of the prepared RLX/NRG NLCs formulations. Moreover, it was discovered that the prepared NLCs formulations had positive electrical charges on their surfaces, which can be displayed as ZP (ζ), with values of +25.9 mV. The values of ZP in Figure 6B indicate that the prepared RLX/NRG NLCs have a relatively long duration of stability.

#### 3.3.4. Surface Morphology Study

Representative microscopic images, as shown in Figure 6C,D, indicate that the RLX/NRG NLC particles were almost spherical, with a homogenous size distribution. The sizes of the RLX/NRG NLCs assessed by TEM and SEM image analysis were identical to the DLS data. According to the results of the TEM and SEM examinations, the nanoparticles were well-segregated one from another, indicating that no aggregation was taking place. Additionally, the TEM and SEM images confirmed the nanosize (<200 nm) of the prepared RLX/NRG NLCs, confirming the result obtained using the zeta sizer.

#### 3.3.5. Determination of RLX and NRG Entrapment Efficiency

The entrapment efficiencies of the RLX/NRG NLCs were estimated to be between 93.32% and 77.23% for RLX and between 88.81% and 72.29% for NRG. Different lipid and surfactant concentrations, as well as the drugs’ lipophilicity, were responsible for the observed variances in these properties.

#### 3.3.6. In Vitro Drug Release Study

**Figure 7:**
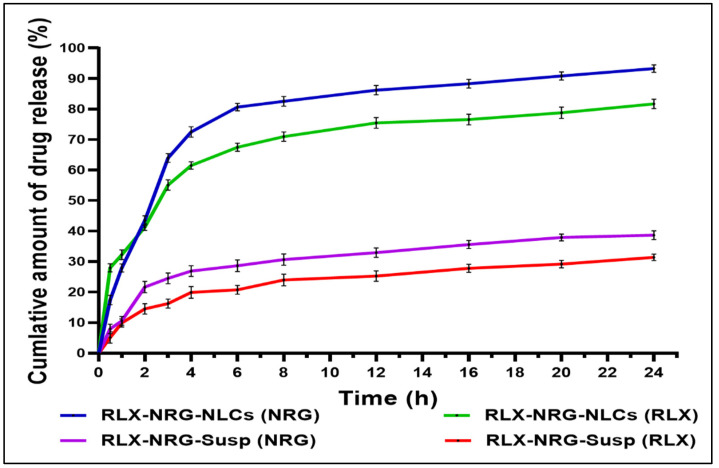
In vitro drug release profiles of RLX and NRG in their combinations in NLCs and suspension. Values expressed as the means ± SD (*n* = 3).

In vitro release of RLX and NRG from the RLX/NRG suspension and RLX/NRG NLCs in HCl (0.1 N, pH 1.2) and the phosphate buffer (pH 6.8) is shown in Figure 7. In the acidic media, RLX and NRG release from the RLX/NRG NLCs was 41% and 43% within 2 h, respectively. In comparison, the RLX and NRG release from the RLX/NRG suspension was just 21% and 14% within 2 h, respectively. The in vitro cumulative release at pH 6.8 revealed that the release of RLX and NRG from the RLX/NRG NLCs was 81% and 93% after 24 h, respectively, whereas the cumulative release of RLX and NRG from their combined suspensions after 24 h was about 31.4% and 38.6%, respectively. The NLCs formulation had an imperfect solid–liquid lipid mixture arrangement, resulting in a 4 h initial drug release followed by a 20 h sustained release. The drugs incorporated in a solid lipid matrix may exhibit a long and delayed release time, whereas the drug in a liquid lipid or free drugs unloaded to the lipid matrix of an NLC may have an immediate release. Moreover, to investigate the drug release mechanism from RLX/NRG NLCs, the release profile was fitted into a variety of release kinetic models to study the mechanism of drug release (Supplementary Figures S2 and S3). The first-order model was found to be the most suitable model for RLX and NRG, with the highest R^2^ values of 0.8708 and 0.8602, respectively (Supplementary Table S6). The kinetics of the first-order release revealed that the release of a drug from the lipid matrix is represented by drug dissolution and diffusion through the porous medium that depends on the drug concentration. The Korsmeyer equation was used to develop the mechanism of RLX and NRG release from the optimized RLX/NRG NLCs by computing the *n*-value from the linear part of the plotted curve. The *n*-values were 0.5822 and 0.6938, showing that the developed RLX/NRG NLCs displayed non-Fickian behavior with erosion and diffusion release, respectively.

#### 3.3.7. Antioxidant Activity

**Figure 8:**
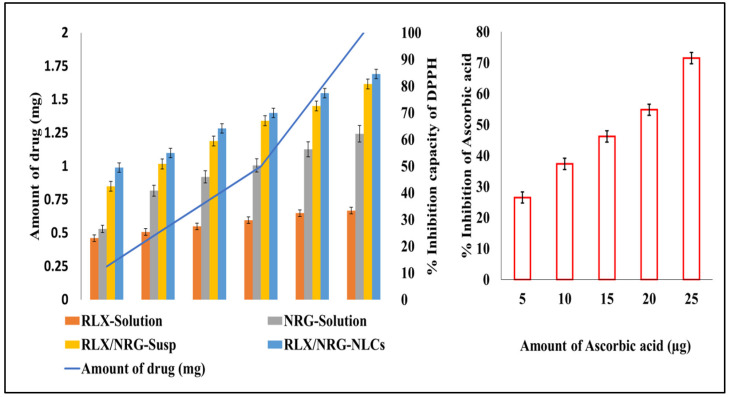
In vitro antioxidant activities of RLX, NRG, RLX/NRG, RLX/NRG NLCs, and ascorbic acid. The results are expressed as the means ± SD (*n* = 3).

The free radical scavenging activity of RLX, NRG, RLX/NRG solution, and RLX/NRG NLCs at different concentrations was determined. Ascorbic acid was included as a standard for radical scavenging ability. Scavenging of the DPPH radical was estimated and is shown in Figure 8. The scavenging activity of DPPH was found to be maximum (84.56%, 80.82%, 62.17%, and 33.39%) at 2 mg of RLX/NRG NLCs, RLX/NRG solution, RLX, and NRG, respectively. The ethanolic solution of the combination of RLX and NRG showed more antioxidant activity than its respective non-combined solution. The slight nonsignificant increase in the antioxidant activity of RLX/NRG NLCs compared to the RLX/NRG solution was attributed to the inclusion of excipients. The IC50 of RLX, NRG, RLX/NRG solution, and RLX/NRG NLCs was calculated to be 4.34 mg, 1.14 mg, 0.45 mg, and 0.19 mg, respectively, while the IC50 of ascorbic acid was 16.22 µg.

#### 3.3.8. Ex Vivo Intestinal Permeation Study

**Figure 9:**
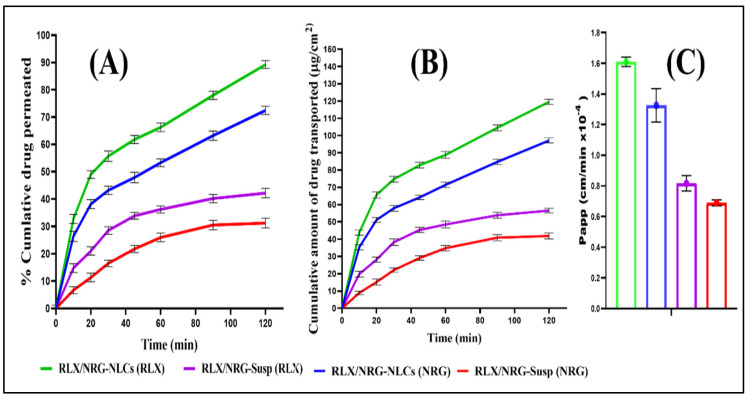
Ex vivo permeation study displaying (**A**) the cumulative drug permeation versus time, (**B**) the cumulative amount of drug transported (μg/cm^2^) versus time, (**C**) the P_app_ for RLX and NRG from the plain RLX/NRG suspension and the RLX/NRG NLCs formulation.

The ex vivo intestinal permeation investigation was used to assess the intestinal permeation and transport of RLX and NRG from RLX/NRG NLCs, which were then compared to the intestinal permeation and transport of the respective suspensions [64]. The cumulative amount of RLX and NRG permeating the intestinal wall to Tyrode’s buffer from RLX/NRG NLCs after 120 min was found to be 89.18 ± 1.89% and 72.45 ± 2.51%, respectively, while in the case of the RLX/NRG suspension, it was found to be 42.16 ± 2.21%, and 31.2 ± 1.28% for RLX and NRG (Figure 9A). Hence, RLX/NRG NLCs exhibited nearly 2.3 and 2.1-fold improvement in the permeability profiles of RLX and NRG vis-à-vis RLX/NRG suspension. The permeation flux (F) was obtained from the linear part of the curve constructed by plotting the cumulative amount of the drug transported (μg/cm^2^) with respect to time (min) (Figure 9B); this revealed that a greater amount of RLX and NRG was delivered from RLX/NRG NLCs through the intestinal mucosal sac than from the RLX/NRG suspension. The apparent permeability coefficients (P_app_) calculated for RLX and NRG from the RLX/NRG NLCs were found (1.61 × 10^−4^ cm min^−1^ and 1.326 × 10^−4^ cm min^−1^), which were significantly higher (*p* < 0.05) than those calculated for RLX and NRG from the RLX/NRG suspension, which were found to be 0.816 *×* 10^−5^ cm min^−1^ and 0.689 × 10^−4^ cm min^−1^, respectively. There was about a 1.973 and 1.924-fold increment in the P_app_ value of RLX and NRG from the RLX/NRG NLCs as compared to the RLX/NRG suspension (Figure 9C). This considerable enhancement in intestinal absorption could be due to increased dissolution of both RLX and NRG after incorporation into the lipid matrix of NLCs, as well as the nanosize of the lipid nanocarriers, contributing to enhanced permeation through the intestinal wall. Aside from that, the presence of various digestible lipid components of Compritol 888 ATO (esters of behenic acid and glycerides) and a fatty acid (oleic acid) in the form of lipid-based nanocarriers further increases the permeability of the drug when it crosses the intestinal wall. Additionally, the use of surfactants such as Tween 80 and Labrasol may improve drug solubility and penetration into the intestinal wall [65,66]. Furthermore, suppression of the P-gp pump by such surfactants increases drug absorption, which in turn increases the absorption and bioavailability of the drug [67,68,69].

#### 3.3.9. Intestinal Uptake by CLSM

**Figure 10:**
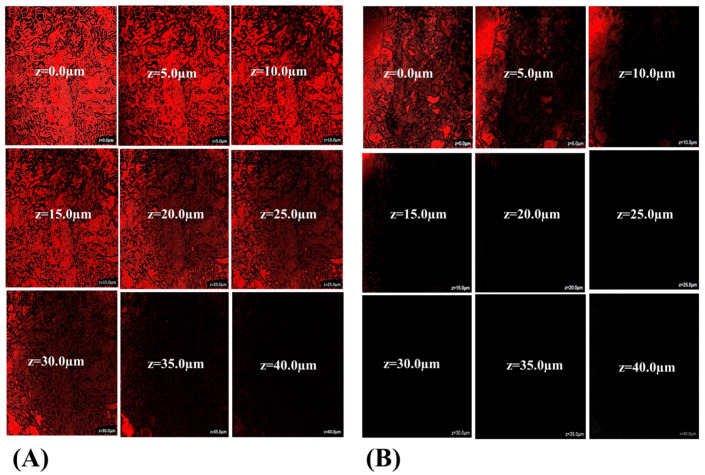
CLSM images of RLX/NRG permeation across the intestinal mucosal membrane treated with (**A**) rhodamine B-labeled RLX/NRG NLCs and (**B**) the rhodamine B-labeled RLX/NRG suspension.

The internalization depth of rhodamine B-labeled RLX/NRG NLCs and RLX/NRG suspension across the intestinal epithelium of rats was detected using CLSM. Figure 10 depicts the depth penetration of fluorescence across the intestinal lumen of a rat. Intense fluorescence was observed up to 35.0 µm (z-axis) with rhodamine B-labeled RLX/NRG NLCs (Figure 10A), while the fluorescence of rhodamine B from the RLX/NRG suspension was detected at 10 µm (z-axis) (Figure 10B). The depth of permeation revealed significant permeation of the drugs from the RLX/NRG NLCs formulation, 3.5-fold higher across the intestine as compared with the RLX/NRG suspension. Internalization of rhodamine B into intestinal cells as depicted by fluorescence intensity demonstrates the significant permeability of the developed RLX/NRG NLCs vis-à-vis RLX/NRG suspension. This result confirms the permeation and transport data from the ex vivo release permeation study discussed in the previous section. The reasons behind this improvement could be explained by the presence of surfactants with high HLB values (15 for Tween 80, 14 for Labrasol) in the RLX/NRG NLCs formulation, which enhances the penetration of RLX/NRG NLCs [70].

#### 3.3.10. In Vivo Acute Toxicity Study

Female Wistar rats were subjected to acute toxicity testing to investigate the potential impact of blank NLCs, RLX/NRG NLCs, RLX NLCs, and NRG NLCs on hematological parameters, visual examination, and histology of critical organs. There were no behavioral abnormalities, acute toxicological effects, or significant weight loss in any of the therapy groups. Furthermore, all the treatment groups showed normal hemoglobin counts, platelet counts, total red blood cell counts, differential white blood cell counts, etc., with no statistically significant (*p* > 0.05) differences in any of these measures (Table 4).

**Figure 11:**
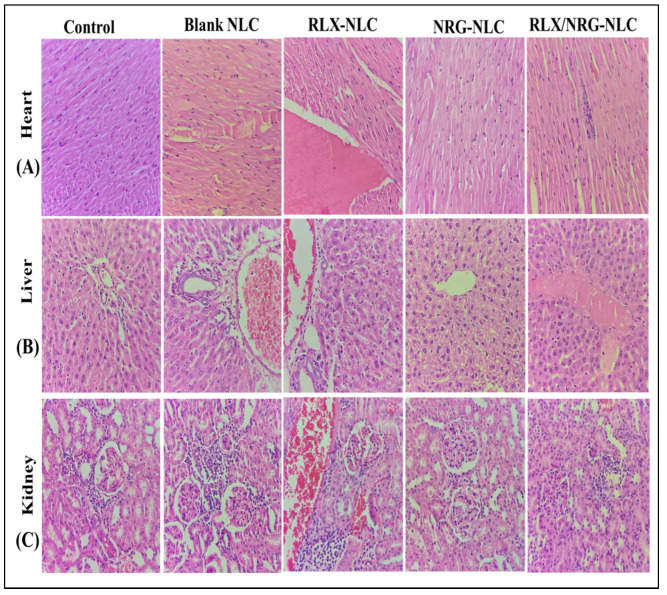
Histological representation ( H & E, 400×) of the (**A**) heart, (**B**) liver, and (**C**) kidney of the rats treated with normal saline, blank NLCs, RLX NLCs, NRG NLCs, and RLX/NRG NLCs.

As shown in Figure 11A, the histological cross-section of the heart from all the treatment groups studied revealed normally ordered and conserved polarity of myocytes grouped in muscular bundles, as seen in normal control rats, with no indication of heart necrosis or bleeding. Multiple interspersed dilated blood vessels are seen, and a single largely dilated vessel filled with plasma and a focal inflammatory cell infiltrate are also seen in the case of the rats treated with RLX NLCs. A mild focal inflammatory cell infiltrate is also seen in the case of the RLX/NRG NLCs group, but it is slightly smaller as compared with the group treated with RLX NLCs. The intercalated disc is also seen in all the treatment groups, similar to the control rats.

As illustrated in Figure 11B, the histological portion of the liver from all the treatment groups investigated demonstrated that the lobular architecture and polarity of the hepatic parenchyma were preserved. Hepatocytes are polygonal in shape and have round to oval nuclei with coarse chromatin and pronounced nucleoli. All the groups showed mild dilatation of sinusoids. Extravasation of RBCs, few dilated blood vessels, and periportal inflammation were also seen in the liver of the rats treated with RLX NLCs and RLX/NRG NLCs. No necrosis was observed in any of the treatment groups.

As illustrated in Figure 11C, the cortex and the medulla of the kidney were visible in histological sections of all the treatment groups studied. The cortex contained multiple intact glomeruli with typical capillary loops, a normal basement membrane, mesangial cells that did not increase in number, and normal mesangial matrices deposition. Many dilated and congested blood vessels along with an inflammatory cell infiltrate were also seen in the RLX NLCs-treated rats, while few were seen in the case of the RLX/NRG NLCs-treated rats and none were seen in the case of the NRG NLCs-, blank NLCs-, and control-treated rats. All of the treated groups had typical proximal convoluted tubules, distal convoluted tubules, the loop of Henle, and the interstitium. There was no evidence of ischemia or necrosis in any of the groups. The results indicated that there was no notable difference between the various treatment groups in the heart, liver, and kidney histological sections. This study reveals the safety of oral NLCs as they did not exhibit toxicity over repetitive use. Moreover, we noticed that incorporation of NRG with RLX into NLCs reduced the toxicity associated with RLX when used alone; this could be attributed to the protective effect of antioxidant activity of NRG.

#### 3.3.11. Storage Stability

**Figure 12:**
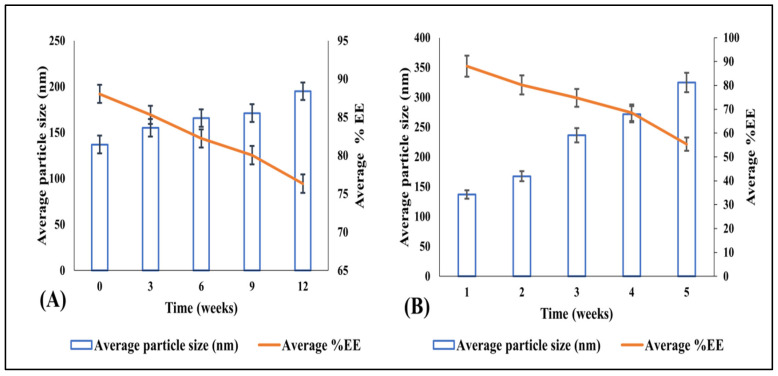
The impact of stability conditions on the particle size and average entrapment efficacy of the optimized lyophilized RLX/NRG-loaded NLCs at 3 months at (**A**) 25 ± 2 °C/60 ± 5% RH and (**B**) 40 ± 2 °C/75 ± 5% RH.

Visual analysis showed no identifiable change in the physical features of the RLX/NRG NLCs, such as color and odor, after six months of storage. Additionally, there was no evidence of dewatering or gas formation in the NLCs. Furthermore, the lyophilized NLCs exhibited a pale yellow free-flowing powder appearance and were easily redispersed. After reconstituting the lyophilized sample to its original volume with double-distilled water, we conducted stability studies to determine its size and entrapment efficacy at various time intervals. Table 5 and Figure 12 show the findings of the storage stability study. However, entrapment efficiency at the accelerated condition was drastically reduced, even though particle size did not vary significantly during testing. The accelerated condition may imply that the drug degrades slowly at this temperature, verifying that the accelerated condition is not an ideal storage environment for lipid-based preparations. As a result, it can be inferred that refrigeration and room conditions (25 ± 2 °C/60 ± 5% RH) are better storage conditions for NLC preparations for a longer period.

During storage at room temperature (25 ± 2 °C/60 ± 5% RH), the particle size increased slightly from 137.04 ± 1.33 to 195.05 ± 2.68 nm, which could be attributed to some particles accumulating during lyophilization. After six months of storage, the average entrapment efficacy of the formulation was 76.32%, showing that some drugs were lost during storage (Figure 12A). This might be due to drug leakage during lyophilization or it could be owing to the loss of some drugs that had adhered to the interface of the NLCs after lyophilization. However, after six months, the entrapment efficacy in the NLCs for RLX/NRG was within reasonable ranges and the size was less than 200 nm. Tween 80 and Labrasol, two stabilizers, work together to make good NLCs that make up for the need for HLB in lipids. Thus, the average particle size was uniformly controlled, and it was easy to redisperse and not form clumps or precipitate while it was in storage.

## 4. Conclusions

The novel RLX/NRG NLCs were successfully prepared using a hot homogenization–ultrasonication technique and systemically optimized using a central composite design approach using three factors at three levels. The optimized RLX/NRG NLCs exhibited a mean particle size of 137.04 nm, the zeta potential of 25.9 mV, entrapment efficiency of 91.77% for RLX and 85.07% for NRG, in vitro release of 81 ± 2.2% from the RLX/NRG NLCs and 31 ± 1.9% from the RLX/NRG suspension for RLX and 93 ± 1.5% from the RLX/NRG NLCs and 38 ± 2.01% from the RLX/NRG suspension for NRG within 24 h. RLX/NRG NLCs exhibited a nearly 2.3 and 2.1-fold improvement in the permeability profiles of RLX and NRG vis-à-vis RLX/NRG suspension. Moreover, the depth of permeation measured by CLSM revealed significant permeation of the drugs from the RLX/NRG NLCs formulation, 3.5-fold higher across the intestine as compared with the RLX/NRG suspension. The in vitro antioxidant activity of the RLX/NRG combination showed a higher antioxidant potential as compared to either drug alone. Moreover, an acute toxicity study confirmed the safety of RLX/NRG NLCs for further use. The lyophilized RLX/NRG NLCs showed satisfactory stability and integrity over three months. Ultimately, our findings suggest that codelivery of raloxifene and naringin using nanostructured lipid carriers could effectively improve therapeutic efficiency and decrease the side effects of the treatment, and further in vivo studies will be conducted.

## Figures and Tables

**Table 1:** Independent variables with their actual levels and dependent variables with their desired outcomes in the CCD.

Independent Variables	High Level (+1)	Medium Level (0)	Low Level (−1)
A = lipid weight (mg)	400	300	200
B = surfactant weight (mg)	200	150	100
C = sonication time (min)	3	2	1
**Dependent variables**		**Desired outcomes**	
Y1 = particle size (nm)		Minimize	
Y2 = polydisperisbility index (PDI)		Minimize	
Y3 = entrapment efficiency of RLX (%)		Maximize	
Y4 = entrapment efficiency of NRG (%)		Maximize	

**Table 2:** ANOVA report for the obtained results in CCD for different experimental runs of RLX/NRG NLCs.

	Factor 1	Factor 2	Factor 3	Response 1		Response 2		Response 3		Response 4	
Run	Lipid (mg)	Surfactant (mg)	Sonication Time (mn)	Particle Size (nm)		PDI		EE%, RLX		EE%, NRG	
				Actual	Predicted	Actual	Predicted	Actual	Predicted	Actual	Predicted
1	−1	0	0	131.6	131.56	0.238	0.2341	86.45	85.95	76.87	78.03
2	1	1	−1	159.38	159.82	0.291	0.2892	85.72	86.09	79.69	80.25
3	0	0	1	**137.12**	**137.04**	**0.266**	**0.2691**	**91.05**	**90.77**	**85.07**	**85.05**
4	0	0	0	143.2	140.73	0.261	0.2620	90.05	90.43	84.21	83.91
5	−1	1	−1	115.6	114.40	0.225	0.2297	84.22	83.88	78.03	77.22
6	0	0	0	141.05	140.73	0.261	0.2620	90.32	90.43	84.08	83.91
7	0	0	0	139.04	140.73	0.262	0.2620	90.16	90.43	84.42	83.91
8	1	0	0	166.03	167.64	0.282	0.2831	91.53	91.71	85.66	85.23
9	−1	1	1	121.7	122.98	0.224	0.2212	86.82	87.42	77.55	77.90
10	−1	−1	1	138.4	137.57	0.244	0.2466	84.72	84.43	78.81	78.07
11	0	0	2	142.15	140.73	0.261	0.2620	89.92	90.43	85.05	83.91
12	0	1	2	135.6	136.26	0.238	0.2369	88.63	88.45	81.01	81.13
13	1	−1	−1	169.02	167.34	0.317	0.3206	91.82	91.31	85.64	85.11
14	1	1	1	164.2	163.02	0.265	0.2662	85.83	85.38	79.73	79.51
15	0	−1	0	146.4	147.32	0.267	0.2653	89.72	89.57	83.03	83.64
16	0	0	0	142.15	140.73	0.259	0.2620	91.05	90.43	83.02	83.91
17	−1	−1	−1	134.44	135.22	0.268	0.2676	77.23	77.76	72.29	72.33
18	1	−1	1	163.5	164.31	0.289	0.2851	93.32	93.74	88.81	89.44
19	0	0	−1	132.6	134.26	0.297	0.2911	87.83	87.79	81.79	82.54
20	0	0	0	139.94	140.73	0.262	0.2620	90.45	90.43	84.15	83.91

**Table 3:** Statistical regression analysis of all the responses for data fitting into different models.

Model	Adjusted R2	Predicted R2	R2	SD	CV%	Adeq. Precision
**Response 1**						
Linear	0.8715	0.8069	0.8918	5.21		
2FI	0.8864	0.7053	0.9223	4.90		
Quadratic	**0.9859**	**0.9342**	**0.9926**	**1.73**	1.21	43.6130
Cubic	0.9860	−0.7498	0.9956	1.72		
**Response 2**						
Linear	0.8524	0.8023	0.8757	0.0090		
2FI	0.8467	0.7585	0.8951	0.0092		
Quadratic	**0.9735**	**0.8204**	**0.9861**	**0.0038**	1.45	36.6952
Cubic	0.9924	−1.2337	0.9976	0.0020		
**Response 3**						
Linear	0.3190	−0.2172	0.4266	3.02		
2FI	0.6125	−1.1119	0.7349	2.27		
Quadratic	**0.9783**	**0.8410**	**0.9886**	**0.5387**	0.6098	41.9474
Cubic	0.9869	−0.1771	0.9959	0.4184		
**Response 4**						
Linear	0.4686	0.1005	0.5525	2.85		
2FI	0.6540	−0.5362	0.7633	2.30		
Quadratic	**0.9520**	**0.7665**	**0.9747**	**0.8581**	1.05	28.2023
Cubic	0.9633	−4.1038	0.9884	0.7501		

**Table 4:** Hematological indicators in the female Wistar rats treated for 14 days with saline, blank NLCs, NRG NLCs, RLX NLCs, and RLX/NRG NLCs (mean ± SD, *n* = 6).

Parameters (Unit)	Control	Blank NLCs	NRG NLCs	RLX NLCs	RLX/NRG NLCs
Hemoglobin (gm/dl)	14.3 ± 1.02	15.8 ± 0.68	12.7 ± 0.5	13.9 ± 0.62	12.2 ± 0.8
TLC (total leucocyte count) (th/cumm)	8.5 ± 0.2	8.4 ± 0.3	7.8 ± 0.2	4.4 ± 1.2	7.6 ± 1.01
Polymorphs (%)	50 ± 3.2	60 ± 2.2	39 ± 1.5	40 ± 1.7	32 ± 2.4
Lymphocytes (%)	45 ± 4.3	35 ± 3.2	52 ± 1.8	52 ± 0.5	61 ± 1.2
Eosinophil (%)	02 ± 0.3	02 ± 0.1	06 ± 0.2	05 ± 0.1	03 ± 0.3
Monocytes (%)	03 ± 0.2	03 ± 0.2	03 ± 0.02	03 ± 0.01	04 ± 0.1
RBC (millions/cumm)	7.3 ± 0.87	6.58 ± 2.3	6.7 ± 1.8	7.9 ± 1.5	6.8 ± 0.6
HCT (%)	50.3 ± 5.3	39.5 ± 2.5	45.6 ± 1.7	47.8 ± 2.1	43.2 ± 1.1
MCV (fl)	68.6 ± 3.3	80.6 ± 3.1	68.1 ± 1.5	60.6 ± 1.1	63.4 ± 0.8
MCH (pg)	19.5 ± 1.2	26.5 ± 2.2	18.9 ± 1.2	17.6 ± 1.4	17.8 ± 0.56
MCHC (g/dl)	28.5 ± 2.1	30.5 ± 1.1	27.7 ± 2.01	29 ± 0.5	28.1 ± 1.05
Platelet count (th/µL)	874 ± 5.3	652 ± 4.3	745 ± 3.2	847 ± 5.3	840 ± 6.31
MPV (fl)	7.1 ± 0.4	8.5 ± 1.1	7.2 ± 0.5	7.6 ± 1.2	8.9 ± 0.5
RDW-CV (%)	15.9 ± 1.2	14.5 ± 1.3	14.8 ± 0.5	16 ± 1.02	13.8 ± 0.7
RDW-SD (fl)	55.8 ± 2.5	45.6 ± 1.2	47.8 ± 2.1	41.5 ± 1.3	47.8 ± 2.2
PCT (%)	0.7 ± 0.2	0.6 ± 0.3	0.7 ± 0.1	0.6 ± 0.2	0.8 ± 0.2
PDW-SD (fl)	15.4 ± 0.3	15.6 ± 0.5	15.4 ± 0.2	15.2 ± 0.1	15.9 ± 0.3

**Table 5:** Stability study data for the optimized RLX/NRG NLCs.

Storage Condition	25 ± 2 °C/60 ± 5% RH			40 ± 2 °C/75 ± 5% RH		
Time (Weeks)	Physical Appearance and Separation	Average Particle Size (nm)	Mean EE% of RLX/NRG ± SD	Physical Appearance and Separation	Average Particle Size (nm)	Mean EE% of RLX/NRG ± SD
0	Pale yellow free-flowing powder/easily re-dispersible powder	137.04	88.06 ± 1.63	Pale yellow free-flowing powder/easily re-dispersible powder	137.04	88.06 ± 1.63
3		155.03	85.32 ± 0.9		167.52	80.25 ± 0.74
6		165.90	82.23 ± 1.25		236.49	74.76 ± 1.82
9		171.22	80.05 ± 1.45		271.42	68.54 ± 1.92
12		195.05	76.32 ± 1.22		325.05	55.34 ± 1.47

## Data Availability

This study did not report any data (all the data are included in the current manuscript).

## Supplementary Materials

The following supporting information can be downloaded at: https://www.mdpi.com/article/10.3390/pharmaceutics14091771/s1, Table S1: Miscibility-based screening of solid lipid and liquid lipid ratios; Table S2: Statistical ANOVA analysis for particle size response by quadratic model; Table S3: Statistical ANOVA analysis for PDI response by quadratic model; Table S4: Statistical ANOVA analysis for EE% RLX response by quadratic model; Table S5: Statistical ANOVA analysis for EE% NRG response by quadratic model; Table S6: Kinetics of release of RLX and NRG from RLX/NRG-NLCs; Figure S1: Predicted vs actual values linear correlation plots for (A) Particle size, (B) PDI and (C,D) % Entrapment Efficiency of RLX/NRG-NLCs; Figure S2: Perturbation correlation plots for (A) Particle size, (B) PDI and (C,D) % Entrapment Efficiency of RLX/NRG-NLCs; Figure S3: Release kinetic study for RLX in the RLX/NRG-NLCs; Figure S4: Release kinetic study of NRG in RLX/NRG-NLCs.

## Abbreviations

RLX: raloxifene, NRG: naringin, CCD: central composite design, CLSM: confocal laser scanning microscopy, CPCSEA: Committee for the Purpose of Control and Supervision of Experiments on Animals, DPPH: dithiobis-2-nitrobenzoic acid, EE%: entrapment efficiency, NLC: nanostructured lipid carrier, RLX/NRG NLC: raloxifene/naringin-loaded nanostructured lipid carrier, ZP: zeta potential, PDI: polydispersity index, SD: standard deviation, TEM: transmission electron microscope, SEM: scanning electron microscopy, FTIR: Fourier-transform infrared spectroscopy, XRD: X-ray diffraction.
