# Supplementary material for: Nanostructured Lipid Carrier-Based Codelivery of Raloxifene and Naringin: Formulation, Optimization, In Vitro, Ex Vivo, In Vivo Assessment, and Acute Toxicity Studies
Source: Pharmaceutics. 2022 Aug 25;14(9):1771. doi: 10.3390/pharmaceutics14091771 (PMC9500671; doi:10.3390/pharmaceutics14091771)
Supplement: Supplementary file 1 [file pharmaceutics-14-01771-s001.zip › pharmaceutics-1838756-supplementary.pdf]

Supplementary Tables

Supplementary Table S1. Miscibility-based screening of solid lipid and liquid lipid ratios.

S.NO.	Solid lipid	Liquid lipid	Interference
1	90	10	No phase separation
2	80	20	No phase separation
3	70	30	No phase separation
4	60	40	No phase separation
5	50	50	Phase separation
6	40	60	Phase separation
7	30	70	Phase separation
8	20	80	Phase separation
9	10	90	Phase separation

Supplementary Table S2. Statistical ANOVA analysis for particle size response by quadratic model.

**Source**	**Sum of Squares**	**df**	**Mean Square**	**F-value**	**p-value**	
Model	3983.35	9	442.59	148.47	< 0.0001	significant
A-Lipid	3254.06	1	3254.06	1091.62	< 0.0001	
B-Surfactant	305.59	1	305.59	102.51	< 0.0001	
C-Sonication time	19.27	1	19.27	6.46	0.0293	
AB	88.44	1	88.44	29.67	0.0003	
AC	14.47	1	14.47	4.85	0.0521	
BC	19.47	1	19.47	6.53	0.0286	
A^2	216.58	1	216.58	72.66	< 0.0001	
B^2	3.09	1	3.09	1.04	0.3328	
C^2	70.98	1	70.98	23.81	0.0006	
Residual	29.81	10	2.98			
Lack of Fit	17.75	5	3.55	1.47	0.3411	not significant
Pure Error	12.06	5	2.41			
Cor Total	4013.16	19				
**Statistical fattiness analysis for particle size response by quadratic model**						
Std. Dev.	1.73		R^2			0.9926
Mean	143.16		Adjusted R^2			0.9859
C.V. %	1.21		Predicted R^2			0.9342
			Adeq Precision			43.6130

Supplementary Table S3. Statistical ANOVA analysis for PDI response by quadratic model.

**Source**	**Sum of Squares**	**df**	**Mean Square**	**F-value**	**p-value**	
Model	0.0104	9	0.0012	78.68	< 0.0001	significant
A-Lipid	0.0060	1	0.0060	409.02	< 0.0001	
B-Surfactant	0.0020	1	0.0020	137.40	< 0.0001	
C-Sonication time	0.0012	1	0.0012	82.45	< 0.0001	
AB	0.0000	1	0.0000	1.44	0.2579	
AC	0.0001	1	0.0001	7.16	0.0232	
BC	0.0001	1	0.0001	5.32	0.0437	
A^2	0.0000	1	0.0000	2.18	0.1708	
B^2	0.0003	1	0.0003	22.30	0.0008	
C^2	0.0009	1	0.0009	61.33	< 0.0001	
Residual	0.0001	10	0.0000			
Lack of Fit	0.0001	5	0.0000	23.46	0.0018	significant

Pure Error	6.000E-06	5	1.200E-06			
Cor Total	0.0105	19				
**Statistical fattiness analysis for PDI response by quadratic model**						
Std. Dev.	0.0038		R^2		0.9861	
Mean	0.2639		Adjusted R^2		0.9735	
C.V. %	1.45		Predicted R^2		0.8204	
			Adeq Precision		36.6952	

Supplementary Table S4. Statistical ANOVA analysis for EE % RLX response by quadratic model.

**Source**	**Sum of Squares**	**df**	**Mean Square**	**F-value**	**p-value**	
Model	250.78	9	27.86	96.00	< 0.0001	significant
A-Lipid	82.83	1	82.83	285.37	< 0.0001	
B-Surfactant	3.12	1	3.12	10.77	0.0083	
C-Sonication time	22.26	1	22.26	76.69	< 0.0001	
AB	64.30	1	64.30	221.53	< 0.0001	
AC	8.99	1	8.99	30.97	0.0002	
BC	4.93	1	4.93	16.98	0.0021	
A^2	7.09	1	7.09	24.43	0.0006	
B^2	5.55	1	5.55	19.13	0.0014	
C^2	3.67	1	3.67	12.66	0.0052	
Residual	2.90	10	0.2903			
Lack of Fit	2.09	5	0.4189	2.59	0.1597	not significant
Pure Error	0.8082	5	0.1616			
Cor Total	0.0105	19				
**Statistical fattiness analysis for RLX % EE response by quadratic model**						
Std. Dev.	0.5387		R^2		0.9886	
Mean	88.34		Adjusted R^2		0.9783	
C.V. %	0.6098		Predicted R^2		0.8410	
			Adeq Precision		41.9474	

Supplementary Table S5. Statistical ANOVA analysis for EE% NRG response by quadratic model.

**Source**	**Sum of Squares**	**df**	**Mean Square**	**F-value**	**p-value**	
Model	283.97	9	31.55	42.85	< 0.0001	significant
A-Lipid	129.46	1	129.46	175.82	< 0.0001	
B-Surfactant	15.80	1	15.80	21.46	0.0009	
C-Sonication time	15.70	1	15.70	21.32	0.0010	
AB	47.58	1	47.58	64.62	< 0.0001	
AC	1.00	1	1.00	1.36	0.2707	
BC	12.83	1	12.83	17.42	0.0019	
A^2	14.36	1	14.36	19.50	0.0013	
B^2	6.44	1	6.44	8.74	0.0144	
C^2	0.0396	1	0.0396	0.0538	0.8213	
Residual	7.36	10	0.7363			
Lack of Fit	5.19	5	1.04	2.40	0.1798	not significant
Pure Error	2.17	5	0.4336			
Cor Total	291.33	19				
**Statistical fattiness analysis for NRG % EE response by quadratic model**						
Std. Dev.	0.8581		R^2		0.9747	
Mean	81.95		Adjusted R^2		0.9520	
C.V. %	1.05		Predicted R^2		0.7665	
			Adeq Precision		28.2023	

Supplementary Table S6. Kinetics of release of RLX and NRG from RLX/NRG-NLCs.

**Drug loaded NLCs**	**Zero order (R^2)**	**First order (R^2)**	**Hixon (R^2)**	**Higuchi (R^2)**	**Peppas**		**Higuchi (R^2)**	**Best fit**
					(R^2)	n		
RAL	0.6273	0.8708	0.7511	0.8519	0.3748	0.5822	0.8519	First order
NAR	0.6105	0.8602	0.7767	0.8327	0.4719	0.6938	0.8327	First order

Supplementry figures

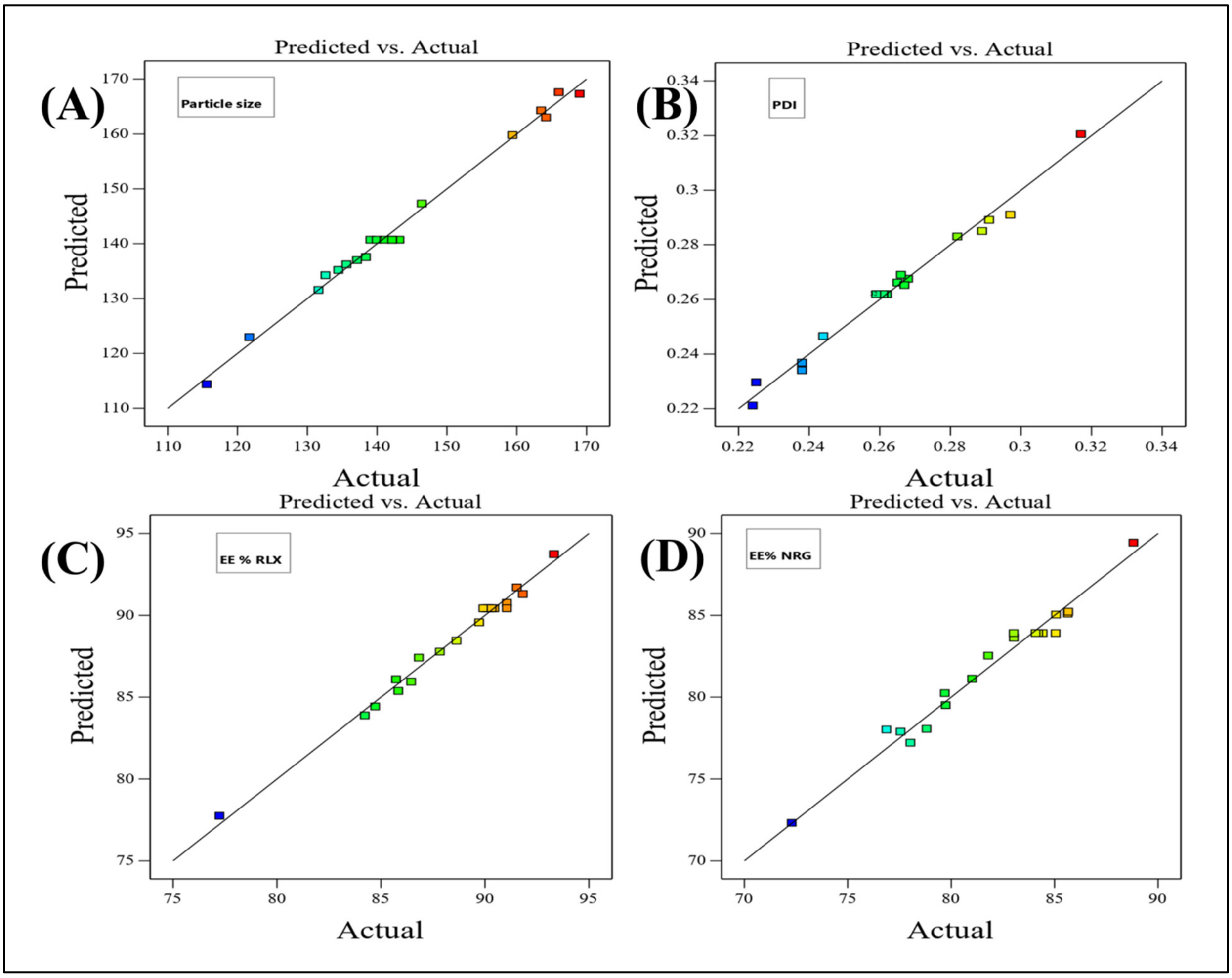

Supplementary Figure S1: Predicted vs actual values linear correlation plots for (**A**) Particle size, (**B**) PDI and (**C**,**D**) % Entrapment Efficiency of RLX/NRG-NLCs.

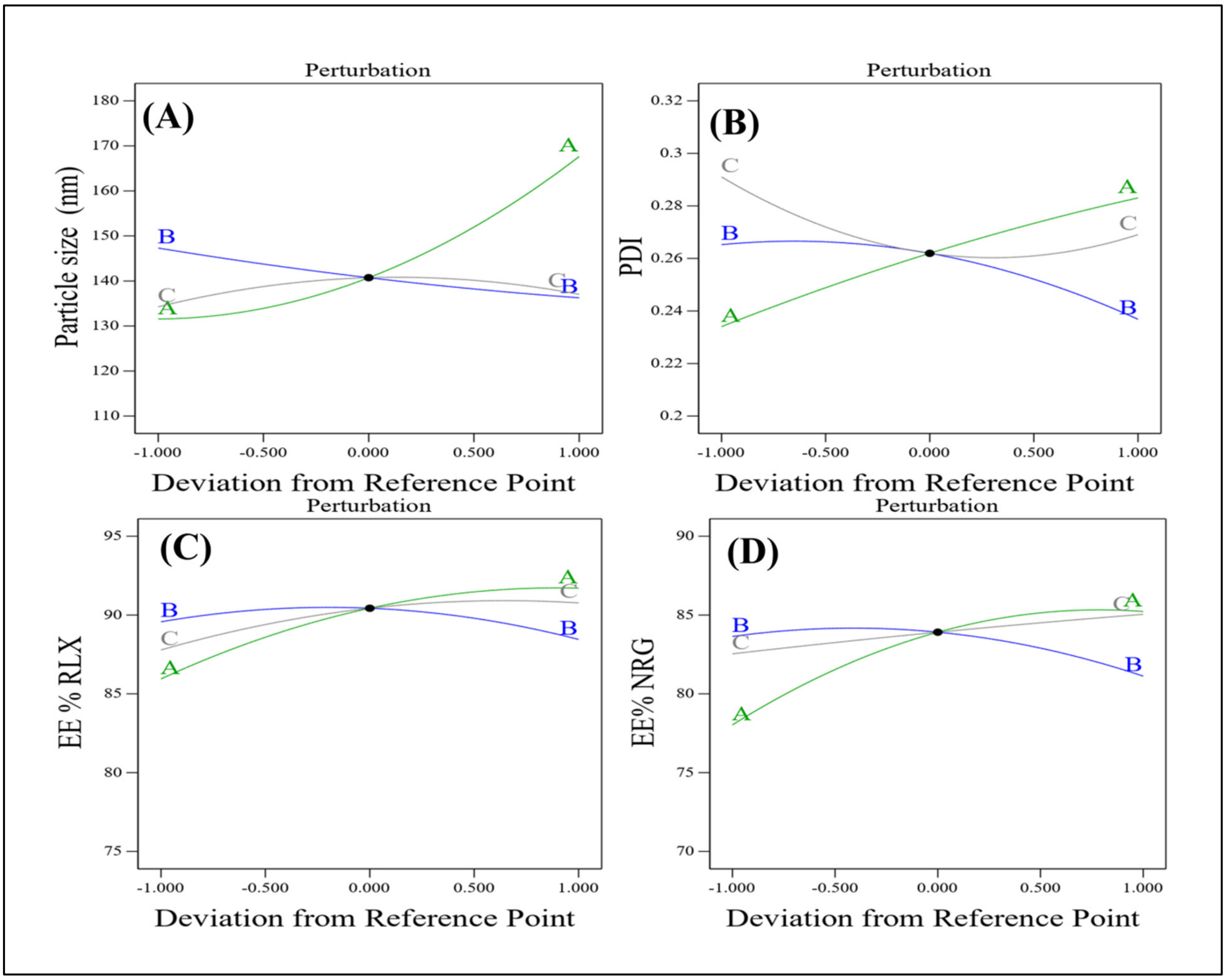

Supplementary Figure S2: Perturbation correlation plots for (**A**) Particle size, (**B**) PDI and (**C**,**D**) % Entrapment Efficiency of RLX/NRG-NLCs.

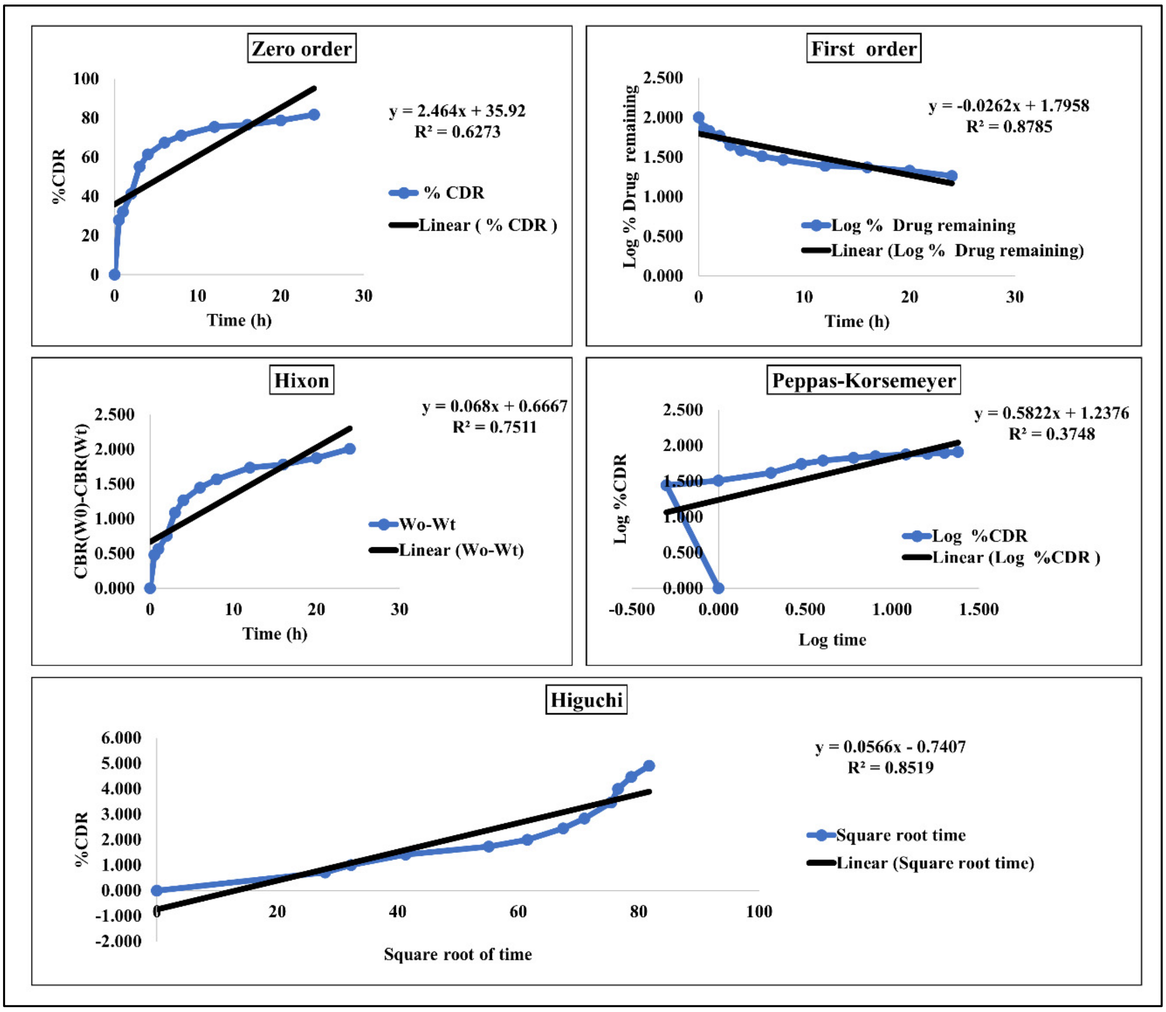

Supplementry Figure S3. Release kinetic study for RLX in the RLX/NRG-NLCs.

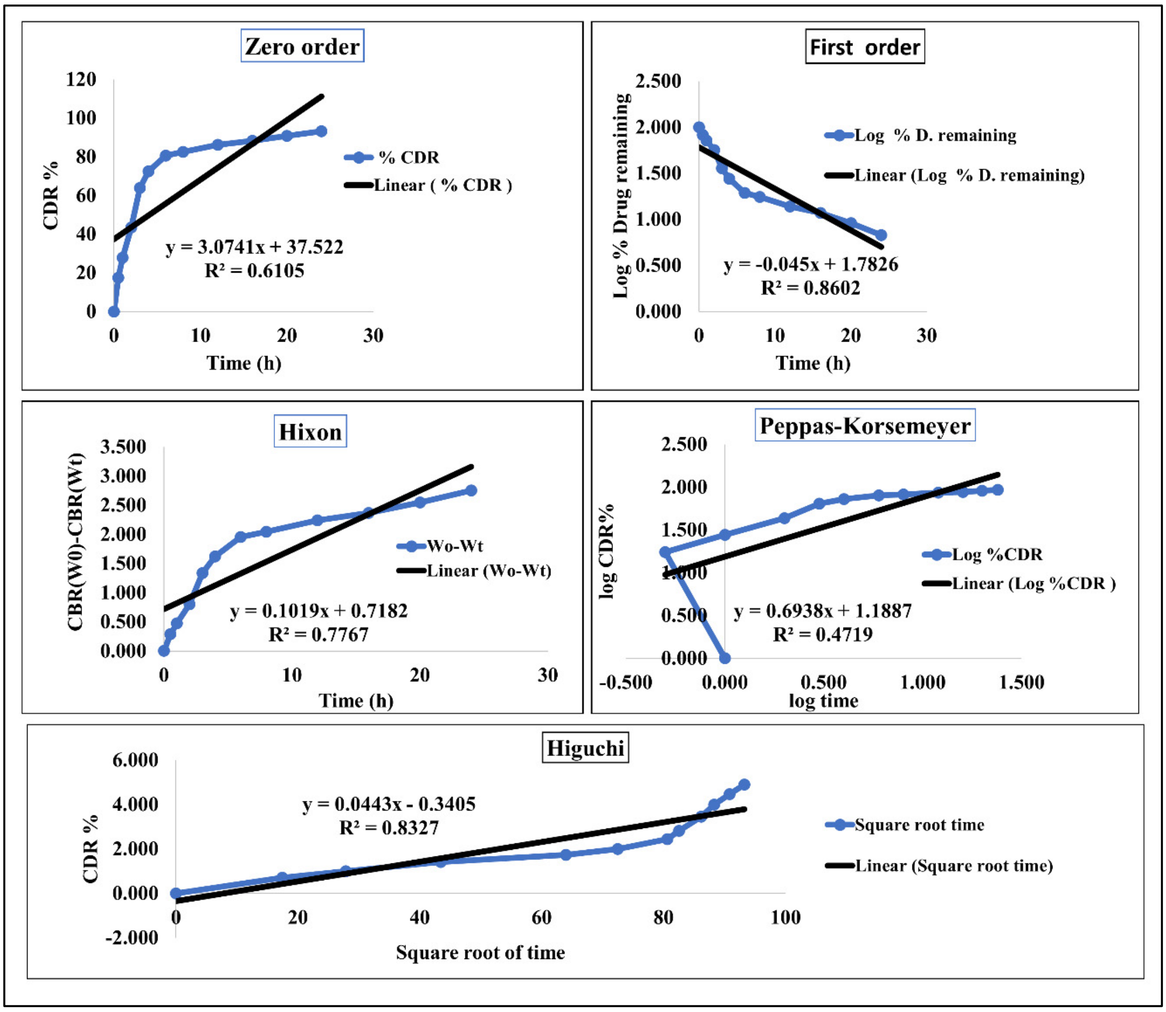

Supplementary Figure S4. Release kinetic study of NRG in RLX/NRG-NLCs.
